# An Updated Functional Annotation of Protein-Coding Genes in the Cucumber Genome

**DOI:** 10.3389/fpls.2018.00325

**Published:** 2018-03-15

**Authors:** Hongtao Song, Kui Lin, Jinglu Hu, Erli Pang

**Affiliations:** ^1^MOE Key Laboratory for Biodiversity Science and Ecological Engineering, College of Life Sciences, Beijing Normal University, Beijing, China; ^2^Graduate School of Information, Production and Systems, Waseda University, Kitakyushu-shi, Japan

**Keywords:** cucumber, gene functional annotation, collinear segments, orthology, protein-coding gene

## Abstract

**Background:** Although the cucumber reference genome and its annotation were published several years ago, the functional annotation of predicted genes, particularly protein-coding genes, still requires further improvement. In general, accurately determining orthologous relationships between genes allows for better and more robust functional assignments of predicted genes. As one of the most reliable strategies, the determination of collinearity information may facilitate reliable orthology inferences among genes from multiple related genomes. Currently, the identification of collinear segments has mainly been based on conservation of gene order and orientation. Over the course of plant genome evolution, various evolutionary events have disrupted or distorted the order of genes along chromosomes, making it difficult to use those genes as genome-wide markers for plant genome comparisons.

**Results:** Using the localized LASTZ/MULTIZ analysis pipeline, we aligned 15 genomes, including cucumber and other related angiosperm plants, and identified a set of genomic segments that are short in length, stable in structure, uniform in distribution and highly conserved across all 15 plants. Compared with protein-coding genes, these conserved segments were more suitable for use as genomic markers for detecting collinear segments among distantly divergent plants. Guided by this set of identified collinear genomic segments, we inferred 94,486 orthologous protein-coding gene pairs (OPPs) between cucumber and 14 other angiosperm species, which were used as proxies for transferring functional terms to cucumber genes from the annotations of the other 14 genomes. In total, 10,885 protein-coding genes were assigned Gene Ontology (GO) terms which was nearly 1,300 more than results collected in Uniprot-proteomic database. Our results showed that annotation accuracy would been improved compared with other existing approaches.

**Conclusions:** In this study, we provided an alternative resource for the functional annotation of predicted cucumber protein-coding genes, which we expect will be beneficial for the cucumber's biological study, accessible from http://cmb.bnu.edu.cn/functional_annotation. Meanwhile, using the cucumber reference genome as a case study, we presented an efficient strategy for transferring gene functional information from previously well-characterized protein-coding genes in model species to newly sequenced or “non-model” plant species.

## Introduction

Cucumber (*Cucumis sativus* L.) (Huang et al., 2009) is an economically important crop as well as a model system for sex determination studies and plant vascular biology (Tanurdzic and Banks, 2004). The whole genome sequence and genome structure annotation of cucumber were published several years ago (Huang et al., 2009; Li et al., 2011). Approximately 23,248 protein-coding genes were predicted in cucumber reference genome, but only a few genes' functions had been verified by experiments (Shang et al., 2014). Recently, the whole genomes of three Cucurbitaceae species have been sequenced and assembled (Huang et al., 2009; Garcia-Mas et al., 2012; Guo et al., 2013). Together with the currently available genomes of other related angiosperms, it provided an opportunity to perform a more accurate functional annotation for these predicted protein-coding genes in cucumber.

Traditional approaches for automatic functional annotation of protein-coding genes in “non-model” species or in newly sequenced genomes rely on homology transfer based on sequence similarity, including Blast2GO (Conesa et al., 2005), identification of conserved domains (Rentzsch and Orengo, 2013) and InterPro2GO (Burge et al., 2012). In addition, OrthoMCL (Li et al., 2003) has been used to identify orthologous relationships between several related genomes using the Bidirectional Best Hits (BBH) strategy and then to transfer annotations of known genes to newly predicted genes. Accurately determining orthologous relationships between genes allows for a better and more robust functional assignment of predicted genes described as the “orthology-function conjecture” (Nehrt et al., 2011). Furthermore, the collinearity information could facilitate reliable orthology inference among multiple related genomes (Zheng et al., 2005). Currently, identification of collinear segments is primarily based on the conservation of genes order (Tang et al., 2008). Unfortunately, many events during plant genome evolution, including whole genome duplications (WGD) (Bowers et al., 2003), reshuffling of short DNA segments by mobile elements and horizontal gene transfer (HGT), have disrupted or distorted the genes order along chromosomes, thus making it difficult to use genes as markers for genome-wide comparisons of plant genomes (Tang et al., 2008). In fact, several comparative genomic studies have demonstrated that smaller units, such as evolutionarily stable domains or segments, are more effective genomic markers than coding genes for whole-genome comparisons (Gabaldón and Koonin, 2013).

In this study, using the cucumber genome as a case study, we annotated functions of the protein-coding genes by integrating information about the collinearity of conserved DNA segments originating from multiple alignments of several related plant genomes.

## Materials and methods

### Data source

In this study, we used three different types of information as detailed in Table 1, including (1) genome sequences for multiple whole-genome alignments, (2) known functional annotations of genes in 14 non-cucumber genomes for functional transfer, and (3) 10 cucumber gene expression datasets used to validate the functional annotation of predicted genes.

Table 1Genome assembly versions, annotation resources, gene expression datasets and download URLs.

**Table:** 

**Species**	**Assembly name**	**Annotation release**	**Release date**	**URL**
**GENOMES DATA SOURCE**				
*Cucumis sativus*	V20	V2	2014/08/08	http://cmb.bnu.edu.cn/Cucumis_sativus_v20/index.html
*Cucumis melo*	ASM31304v1	Release100	2012/10/05	ftp://ftp.ncbi.nlm.nih.gov/genomes/Cucumis_melo
*Citrullus lanatus*	V1	V1	2013/09/21	ftp://www.icugi.org/pub/genome/watermelon
*Vitis vinifera*	12X	Release101	2014/12/10	ftp://ftp.ncbi.nlm.nih.gov/genomes/Vitis_vinifera
*Malus domestica*	MalDomGD1.0	Release100	2012/08/16	ftp://ftp.ncbi.nlm.nih.gov/genomes/Malus_domestica
*Citrus sinensis*	Csi_valencia_1.0	Release100	2012/12/12	ftp://ftp.ncbi.nlm.nih.gov/genomes/Citrus_sinensis
*Populus trichocarpa*	Poptr2_0	Poptr2_0	2013/10/18	ftp://ftp.ncbi.nlm.nih.gov/genomes/Populus_trichocarpa
*Glycine max*	V1.1	Release101	2014/01/07	ftp://ftp.ncbi.nlm.nih.gov/genomes/Glycine_max
*Solanum tuberosum*	SolTub_3.0	Release100	2013/12/12	ftp://ftp.ncbi.nlm.nih.gov/genomes/Solanum_tuberosum
*Arabidopsis thaliana*	TAIR10	TAIR10	2012/08/22	ftp://ftp.ncbi.nlm.nih.gov/genomes/Arabidopsis_thaliana
*Arabidopsis lyrata*	V1.0	V1.0	2014/08/11	ftp://ftp.ncbi.nlm.nih.gov/genomes/Arabidopsis_lyrate
*Brassica rapa*	Brapa_1.0	Release100	2014/09/08	ftp://ftp.ncbi.nlm.nih.gov/genomes/Brassica_rapa
*Setaria italic*	SetariaV1	Release100	2013/06/26	ftp://ftp.ncbi.nlm.nih.gov/genomes/Setaria_italic
*Brachypodium distachyon*	V1.0	Release101	2014/12/14	ftp://ftp.ncbi.nlm.nih.gov/genomes/Bracgypodium
*Oryza brachyantha*	V1.4b	Release100	2014/01/14	ftp://ftp.ncbi.nlm.nih.gov/genomes/Oryza_brachyantha

**Table:** 

**Species**	**Source of database**	**Download date**	**URL**
**GENES FUNCTIONAL ANNOTATION DATA SOURCE**			
*Citrus sinensis*	UniProt_proteome	2016/5/10	http://www.uniprot.org/proteomes/UP000027120
*Glycine max*	UniProt_proteome	2016/5/10	http://www.uniprot.org/proteomes/UP000008827
*Brachypodium*	UniProt_proteome	2016/5/10	http://www.uniprot.org/proteomes/UP000008810
*Setaria italic*	UniProt_proteome	2016/5/10	http://www.uniprot.org/proteomes/UP000004995
*Vitis vinifera*	UniProt_proteome	2016/5/10	http://www.uniprot.org/proteomes/UP000009183
*Arabidopsis lyrata*	UniProt_proteome	2016/5/10	http://www.uniprot.org/proteomes/UP000008694
*Arabidopsis thaliana*	UniProt_proteome	2016/5/10	http://www.uniprot.org/proteomes/UP000078284
*Cucumis sativus*	UniProt_proteome	2016/5/10	http://www.uniprot.org/proteomes/UP000029981
*Solanum tuberosum*	UniProt_proteome	2016/5/10	http://www.uniprot.org/proteomes/UP000011115
*Oryza brachyantha*	UniProt_proteome	2016/5/10	http://www.uniprot.org/proteomes/UP000006038
*Populus trichocarpa*	UniProt_proteome	2016/5/10	http://www.uniprot.org/proteomes/UP000006729
*Arabidopsis thaliana*	TAIR_10	2016/9/30	http://www.arabidopsis.org/download
*Arabidopsis thaliana*	Plant Ontology V1.2	2016/9/30	https://github.com/Planteome/plant-ontology

**Table:** 

**Species**	**Data type**	**Tissues**	**References**	**URL**
**GENE EXPRESSION DATASET**				
*Cucumis sativus*	RNA-seq	leaf, ovary, fertilized ovary, root, stem, male flower, female flower, tendril, base part of tendril	Li et al., 2011	https://www.ncbi.nlm.nih.gov/sra/?term=SRA046916

### Multiple whole-genome alignment

**Figure 1:**
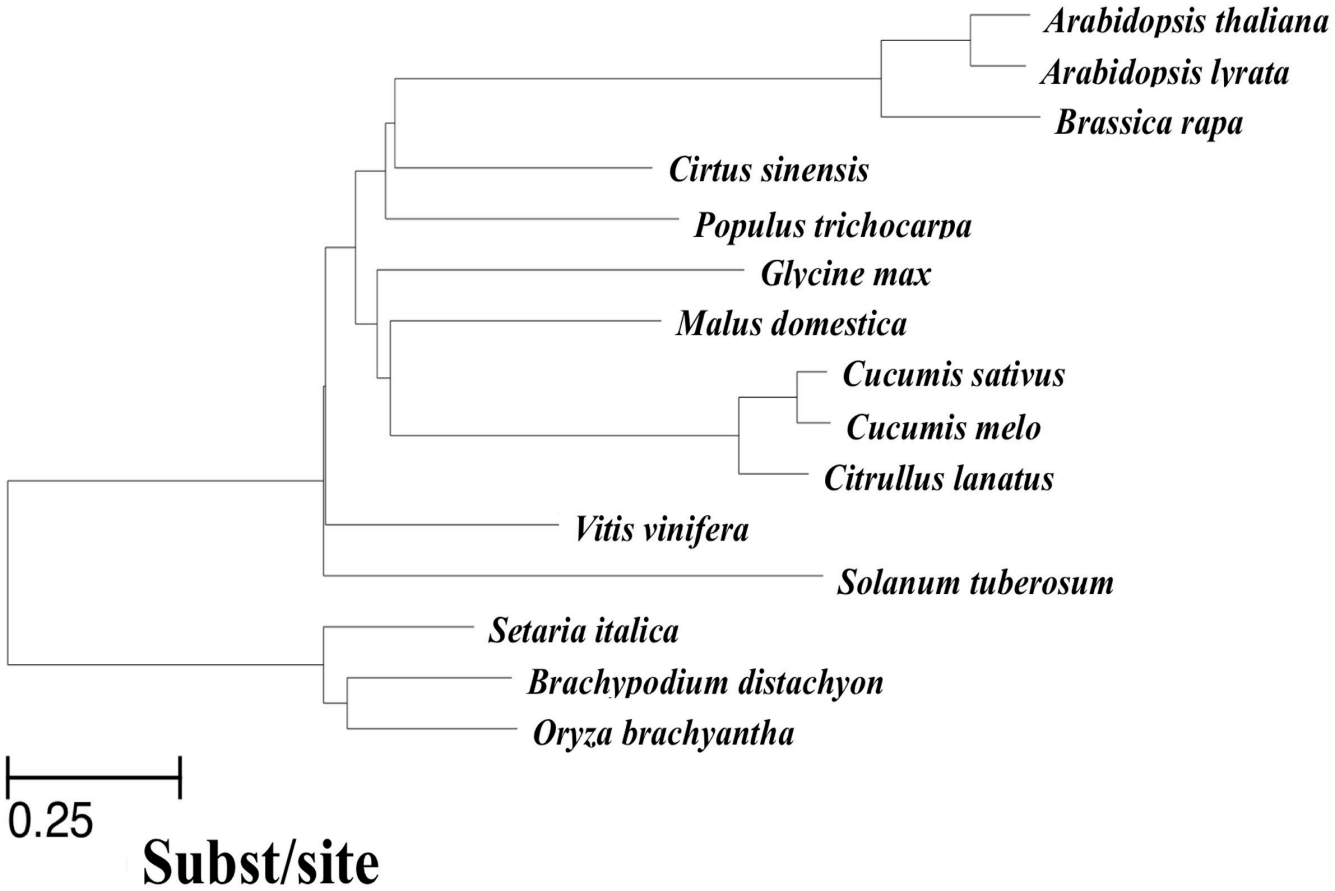
Phylogenetic tree of species included in the 15-way cucumber-based alignment, used to guide MULTIZ merging of pairwise alignments. The neutral tree is based on four-fold degenerate sites sampled from Chr1 ~7 with branch lengths proportional to the indicated scale.

In addition to the cucumber genome, previously published genomes of 14 other related angiosperms were used to perform a multiple whole-genome alignment. To ensure high quality genome assembly and alignment efficiency, we only used sequences that met at least one of the two following requirements: (1) genome sequences were assembled onto chromosomes; (2) N_50_ of the scaffolds was >260 kbp. At the same time, scaffolds shorter than 2,000 bp were removed before alignment. As illustrated in Figure 1, we obtained a phylogenetic tree covering all 15 included species with the evaluated branch lengths by phyloFit (Siepel et al., 2005), and the topology was from an angiosperm super-tree (Davies et al., 2004). To generate a pairwise whole-genome alignment against the cucumber genome (Huang et al., 2009), we used LASTZ (Harris, 2007), a local alignment algorithm optimized for whole-genome alignments, to locally compare the cucumber reference genome with all of the sequences of each query genome. Then, the pairwise alignments were passed through the alignment “chaining” and “netting” pipeline as described by Kent et al. (2003) to ensure that each base of the reference genome was aligned with at most one base in each other genome, using synteny to guide the selection procedure. The resulting pairwise alignments of each query genome to the cucumber reference were joined using MULTIZ (Blanchette et al., 2004), guided by the phylogenetic tree topology in Figure 1. The 15-way multiple alignments can be viewed as a series of conserved blocks that exist in all 15 species that contain the best match within the cucumber reference. We termed these 15-way blocks conserved across 15 angiosperms genomes as the “multiple alignment anchors” (MAAs).

### Collinearity detection among 15 angiosperms plant genomes

Two different types of genomic markers, including MAAs identified by multiple alignments and a set of protein-coding gene families constructed by OrthoMCL (Li et al., 2003), were used individually to detect positional orthologous regions (also known as collinear segments) among the 15 genomes using the i-ADHoRe v3.0 (Proost et al., 2012) program. The running parameters for i-ADHoRe were set as follows: “alignment_method = gg4, anchor_points = 3, gap_size = (MAAs-based as 30, protein-based as 10), prob_cutoff = 0.01, level_2_only = false”. First, i-ADHoRe identified homologous regions (segments) between two genomes containing at least three homologous genes (“anchor points”), with the anchor points separated by at most 10 non-homologous genes or 30 MAAs (“gap_size”). Then, two types of collinear segments between cucumber and the other 14 angiosperm genomes were identified at the n-way and 2way-d levels using in-house Perl scripts based on the i-ADHoRe raw results. The *n*-way (*n*∈{3,4, …, 15}) collinear segments indicate the group of species consisting of cucumber and the other species that were sequentially incorporated based on the topology of the tree in Figure 1, with cucumber as the origin. These segments represent the multiple species level of collinear segments. Each 2way-*d* (*d*∈{2, 3, …, 15}, where *d* is the species index) collinear segment represented a pairwise alignment with one of the 14 non-cucumber species indexed by *d*, where *d* was incremented with the degree of divergence from cucumber according to the phylogenetic tree (Figure 1). Thus, the index *d* (*d*∈{2, 3, …, 15}) represented, in ascending order, *Cucumis melo, Citrullus lanatus, Malus domestica, Glycine max, Populus trichocarpa, Citrus sinensis, Brassica rapa, Arabidopsis thaliana, Arabidopsis lyrata, Vitis vinifera, Solanum tuberosum, Setaria italica, Brachypodium distachyon*, and *Oryza brachyantha*.

### Orthology-inference rule

Based on the collinear segments among cucumber and the other angiosperms plants identified by using MAAs as markers, g_1_ and g_2_ (protein-coding genes from species 1 and 2, respectively) were designated as an orthologous protein-coding gene pairs (OPPs) if they met two essential conditions: (i) g_1_ and g_2_ shared certain sequence similarity (all-vs-all BLASTP, *E*-value = 1E-05); (ii) at least 50% of the length of the g_1_ and g_2_ sequences were located in the same collinear segment. We also applied two optional conditions: (iii) g_1_ and g_2_ were BBH (all-vs-all BLASTP, *E*-value = 1E-05); (iv) g_1_ and g_2_ also met condition (ii) when collinear segments were identified using protein-coding genes as genomic markers.

For each OPP, we calculated an orthologous pair support score (OPSS) from three parameters as follows: (i) the M-score, indicating the degree of the MAAs-based collinear segment (2~15-way) supporting the orthologous relationship; (ii) the P-score, with weight 0.5, indicating the degree of the protein-based collinear segment (2~15-way) supporting the orthologous relationship; and (iii) the B-score indicating the BBH relationship (0: not BBH; 2: BBH).

$$\begin{array}{l} \text{OPSS}\left({\text{g}}_{\text{a}}\text{-}{\text{g}}_{\text{b}}\right)=\text{M-score}+\text{P-score}\times 0.5+\text{B-score} \end{array}$$

### Six existing pipelines for gene functional annotation

To evaluate the quality of the functional annotations from our collinearity-based pipeline, we performed analyses with 6 other commonly-used annotation pipelines, using default parameters, including Blast2GO (Conesa et al., 2005), OrthoMCL (Li et al., 2003), InterPro2GO (Jones et al., 2014), Trinotate-Blast (Grabherr et al., 2011), Trinotate-Pfam (Grabherr et al., 2011), and the UniProt resource, as shown in Table 2. The detailed pipeline parameters settings and procedures were described in the Supplementary Material.

**Table 2:** Six commonly-used pipelines for genome-wide functional annotation.

**Pipeline**	**Functional resources**	**Online URL or Ref**.
Blast2GO	SwissProt database (release_2013-05)	Conesa et al., 2005
OrthoMCL	UniProt database (release_2013-07)	Li et al., 2003
InterProScan	InterProScan-5.3-46.0	Jones et al., 2014
Trinotate-blast	Trinotate.20140708.swissprot.sqlite	http://trinotate.github.io and (Grabherr et al., 2011)
Trinotate-pfam	Trinotate.20140708.swissprot.sqlite	http://trinotate.github.io and (Grabherr et al., 2011)
UniProt resource	UniProt-reference-proteomes	http://www.uniprot.org/proteomes

### Comparison of the different annotation results

To assess the similarity of the different annotation results, we drew on the pipeline comparison strategy from Amar (Amar et al., 2014). We described each annotation result as a triplet (P, G, GO), where P represents the set of all of the annotated gene-GO term pairs, G represents the set of genes covered by P, and GO is the set of GO terms covered by P. Given the two annotation results from pipelines A = (P_A_, G_A_, GO_A_) and B = (P_B_, G_B_, GO_B_), we used three types of similarity indices for comparisons: (i) the Jaccard coefficient (Jaccard, 1912) between P_A_ and P_B_, which was calculated as the ratio of the intersection of P_A_ and P_B_ to the union of P_A_ and P_B_, was used to measure the degree of overlap between two annotation results. This is a structure-free index since it does not consider the hierarchical structure of GO, (ii) the Jaccard coefficient between G_A_ and G_B_ was used to measure the tendency of pipelines A and B to annotate the same gene set and is a structure-free index, (iii) the semantic similarity of the same gene annotated by GO_A_ and GO_B_ was calculated by Wang's method (Wang et al., 2007) as a structure-based index considering the parent-child inheritance relationships of GO. The final semantic similarity score for pipelines A and B was calculated as the average of their gene-wise semantic similarity scores.

### RNA-seq data processing and gene co-expression calculation

Guided by the cucumber reference genome, the RNA-seq datasets from ten cucumber tissues (Li et al., 2011) were processed by the traditional Tophat (Trapnell et al., 2009) and Cufflinks (Trapnell et al., 2010) pipelines to obtain an FPKM (Fragments Per Kilobase of Transcript Per Million Mapped Reads) value for each gene. Given a gene set U associated with a specific GO term and a gene expression matrix X with genes as rows and the ten tissues as columns, Pearson correlation coefficients were calculated for all of the pairs of genes in U using their expression profiles in X. To evaluate whether the correlations in U tend to be higher than expected by chance, the correlation of randomly-sampled gene pairs in X was calculated to obtain the distribution of random correlation scores. Then, the Kolmogorov-Smirnov test (KS test) was used to compare the observed correlation scores of U to the random correlation scores. To improve robustness, this process was repeated 100 times for each gene set U, and the mean *p*-value was used as the final *p*-value for that specific GO term.

## Results

### Alignment of 14 angiosperm genomes to the cucumber reference genome

The popular LASTZ (Harris, 2007)/MULTIZ (Blanchette et al., 2004) framework was used for the whole genome alignment. The dataset of 15 whole genome sequences spanned most of the angiosperm phylogeny, with representatives from three monocots [*S. italica* (Bennetzen et al., 2012), *B. distachyon* (International Brachypodium, 2010), and *Oryza sativa* (Chen et al., 2013)], as well as 12 eudicots, including 3 Cucurbitaceaes [*C. sativus* (Huang et al., 2009), *C. melo* (Garcia-Mas et al., 2012), and *C. lanatus* (Guo et al., 2013)], 2 Fabidaes [*G. max* (Schmutz et al., 2010) and *M. domestica* (Velasco et al., 2010)], 5 Malvidaes [*A. thaliana* (Swarbreck et al., 2008), *A. lyrata* (Hu et al., 2011), *B. rapa* (Wang et al., 2011), *C. sinensis* (Xu et al., 2013) and *P. trichocarpa* (Tuskan et al., 2006)], 1 Solanaceae [*S. tuberosum* (Potato Genome Sequencing et al., 2011)], and 1 Vitaceae [*V. vinifera* (Jaillon et al., 2007)].

**Figure 2:**
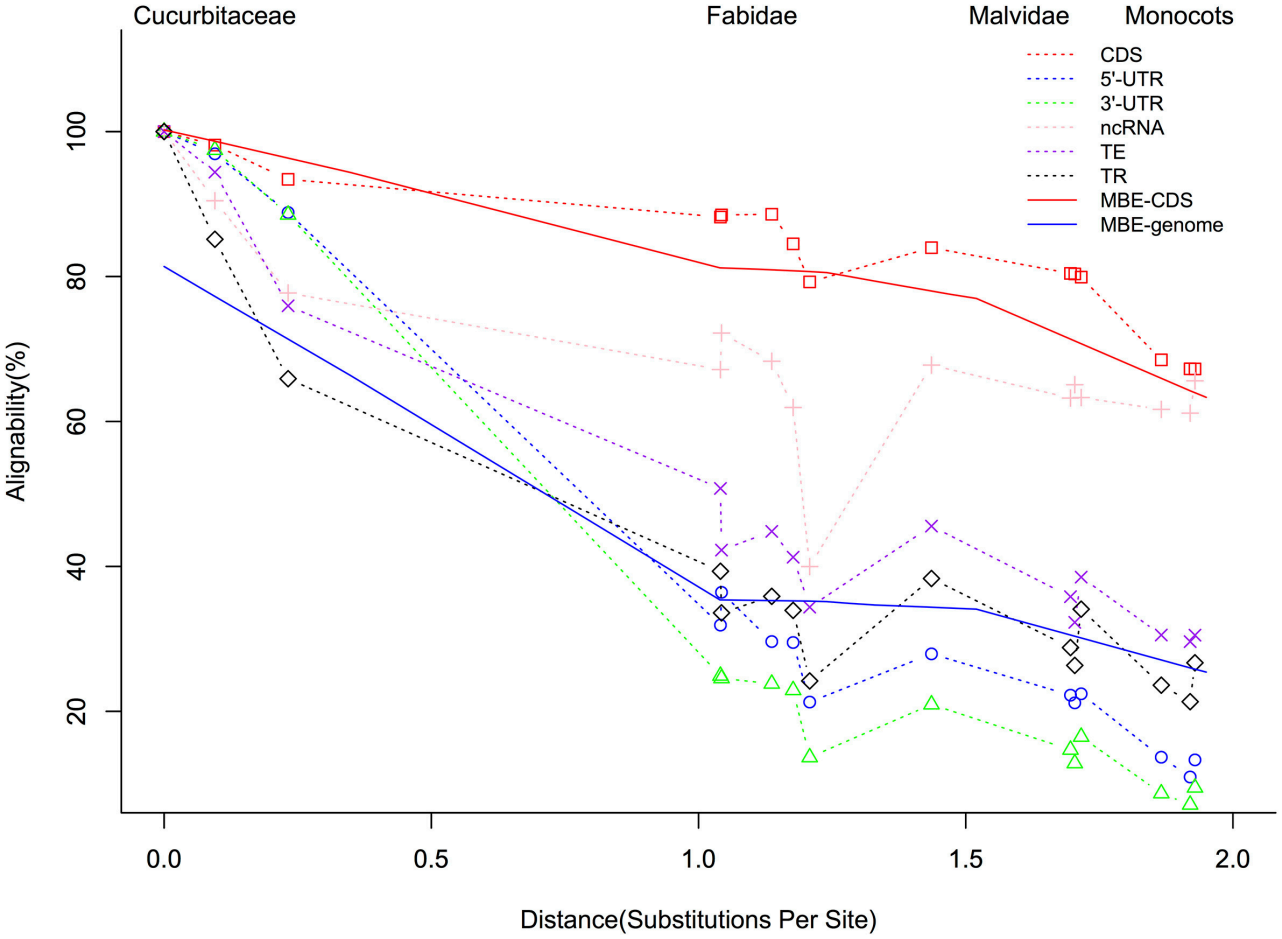
Alignment of *Cucumis sativus* genome features with corresponding features in species at increasing phylogenetic distances. The solid red line indicates the genome alignability while the blue dashed line represents previously described CDS regions (Hupalo and Kern, 2013); CDS, coding sequences; UTR, untranslated region; ncRNA, noncoding RNA; TE, transposable elements; TR, tandem repeats. MBE indicates the results from one previous study (Hupalo and Kern, 2013) published in molecular biology and evolution. “Substitutions per Site” lists the divergence from cucumber based on the phylogeny in Figure 1.

The summary of the pairwise alignment is presented in Table 3, in which the species were ordered by the degree of divergence from cucumber based on the phylogenetic tree (Figure 1). The topology of tree was derived from a previously published angiosperm super-tree (Davies et al., 2004) and the branches were evaluated by phyloFit (Siepel et al., 2005). Although there were differences in genome size, sequencing quality and assembly quality, we observed a trend in which the whole genome alignability decreased with increasing divergence from cucumber, ranging from 91.28% (with *C. melo*) to 24.46% (with *O. sativa*). For coding regions, the alignability ratios were much higher, ranging from 98.13% (with *C. melo*) to 67.26% (with *O. sativa*). In addition, similar patterns were observed when calculating the alignability ratios with different genomic features, as illustrated in Figure 2. Additionally, even when using different reference genomes and including different species for multiple alignments (Hupalo and Kern, 2013), the genome alignability ratios showed a similar decreasing pattern with increasing levels of divergence, as shown in Figure 2.

**Table 3:** Species and alignment coverage in 15-way comparison with cucumber as reference.

**Species**	**Nucleotides (Mbp)**	**References**	**Total align (%)**	**CDS^a^ align (%)**	**Subs/site^b^**
*Cucumis sativus*	188	Huang et al., 2009	–	–	0
*Cucumis melo*	375	Garcia-Mas et al., 2012	91.28	98.13	0.095
*Citrullus lanatus*	339	Guo et al., 2013	75.26	93.41	0.232
*Vitis vinifera*	471	Jaillon et al., 2007	42.76	88.21	1.041
*Malus domestica*	1,874	Velasco et al., 2010	36.94	88.50	1.043
*Citrus sinensis*	318	Xu et al., 2013	38.72	88.59	1.137
*Populus trichocarpa*	298	Tuskan et al., 2006	37.35	84.51	1.177
*Glycine max*	943	Schmutz et al., 2010	27.26	79.26	1.208
*Solanum tuberosum*	686	Potato Genome Sequencing et al., 2011	38.11	83.98	1.436
*Arabidopsis thaliana*	116	Swarbreck et al., 2008	31.04	80.43	1.696
*Arabidopsis lyrata*	201	Hu et al., 2011	28.18	80.36	1.704
*Brassica rapa*	281	Wang et al., 2011	34.98	79.92	1.716
*Setaria italic*	393	Bennetzen et al., 2012	22.99	68.50	1.866
*Brachypodium distachyon*	264	International Brachypodium, 2010	20.67	67.26	1.920
*Oryza brachyantha*	252	Chen et al., 2013	24.46	67.25	1.929

a *CDS as coding sequence*.

b *Subs/Site was evaluated based on four-fold degenerate sites sampled from Chr1~7 with branch lengths by phyloFit (Siepel et al., 2005)*.

### Multiple alignment anchors conserved across all 15 plant species

**Figure 3:**
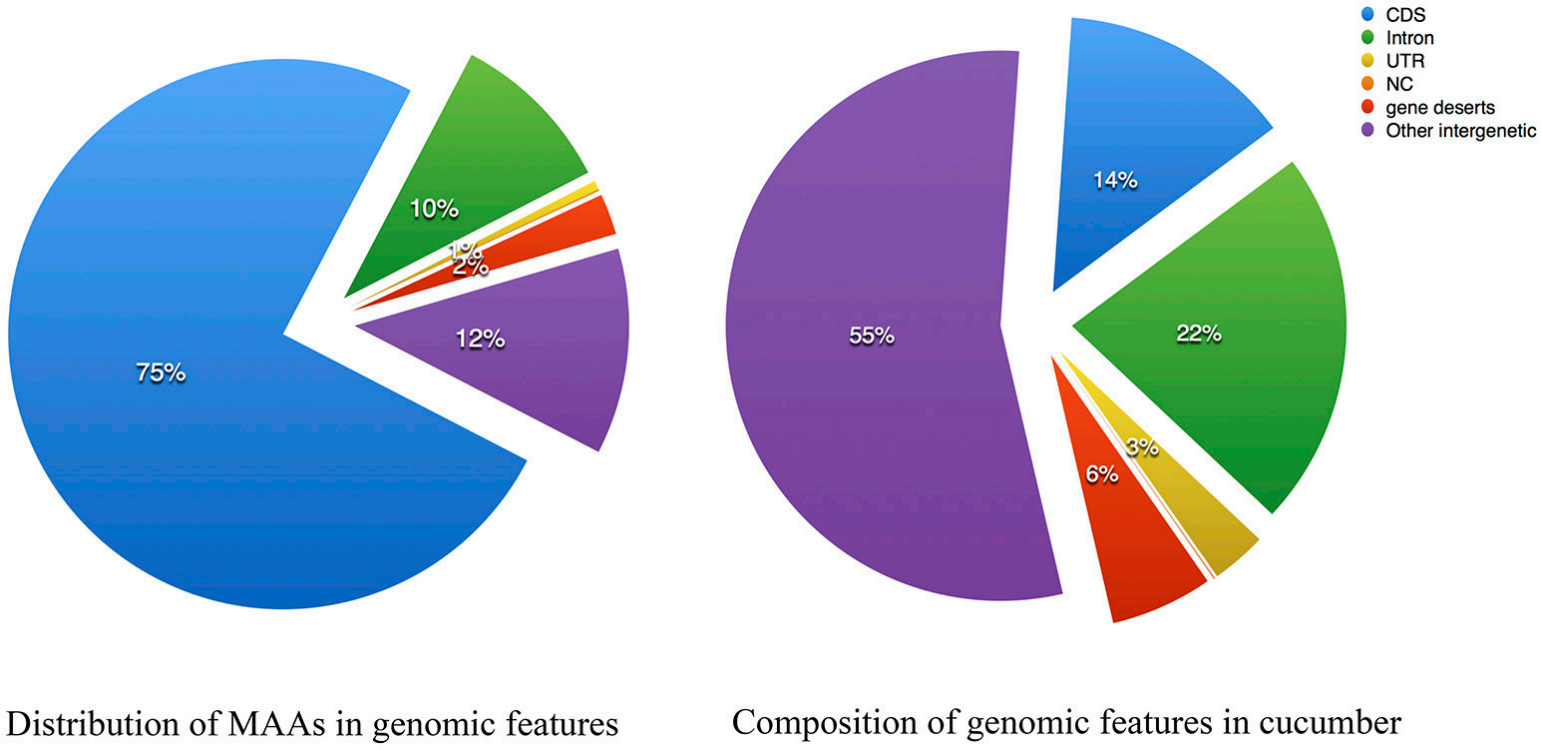
Multiple alignment anchors composition compared with the genome features of cucumber. **(Left)** Composition of Multiple Alignment Anchors; **(Right)** Composition of *Cucumis sativus* V2. CDS, coding sequence; NC, noncoding RNA; UTR, untranslated region, gene deserts: intergenic length between two adjacent genes > 30 Kbp.

The 15-way multiple alignments can be viewed as a series of conserved blocks that exist in all 15 species that contain the best match within the cucumber reference. We termed these 15-way blocks conserved across 15 angiosperms genomes as the “multiple alignment anchors” (MAAs), which can potentially be used as genome-wide markers for detecting genomic homology. The rationale for finding MAAs is that due to long enough divergence time any identified regions of sequence similarity are the result of purifying selection rather than neutral carryover. With the cucumber genome as a reference, we assembled the largest comparative genomic dataset in plants to date using whole-genome sequences spanning the breadth of flowering plants. In total, 138,893 MAAs were identified by our local LASTZ/MULTIZ pipeline. The distribution of the MAAs lengths from chromosome 1~7 is shown in Figure S1. The MAAs ranged in length from 1 to 3,513 bp, with a mean length of 115 bp and a median of 83 bp as showed in Table S3 with cucumber as reference. In total, MAAs covered approximately 8.13% of the cucumber reference genome, which was similar to the proportion of conserved elements in the human genome (3–8%) estimated by (Siepel et al., 2005). The normal composition of genomic features in cucumber was illustrated in Figure 3 and served as a reference to which the MAAs could be compared. The distribution of MAAs in genomic features as illustrated in Figure 3 could be contrasted with the normal distribution, revealing an expansion in the proportion of protein-coding sequence. Meanwhile, inter-genic regions and introns also contained many MAAs, suggesting a substantial number of sequences with undiscovered functionality in the genomes of cucumber and other plants (Hupalo and Kern, 2013).

The identified MAAs were more numerous, shorter in length and more widely distributed than protein-coding genes (Figure S2). Interestingly, the GC content of MAAs was significantly (*P*-value < 2.2E-16) higher than the genome-wide average of cucumber (Figure S3). Previous studies have shown that the GC content was closely related to DNA structural stability and nucleosome formation (Jansen and Verstrepen, 2011), which implies that the identified MAAs may be from stable, potentially functional regions of the genome. Evaluation of the distribution of genomic markers across the cucumber genome by KS test (Figure S4) showed that MAAs were more uniformly distributed than protein-coding genes.

### Atlas of collinear segments between cucumber and other angiosperms inferred by MAAs

i-ADHoRe (Proost et al., 2012) was used to infer collinear regions among 15 angiosperm genomes using two different types of genome-wide markers: the MAAs and protein-coding gene families constructed by OrthoMCL (Li et al., 2003). In total, 1,983 n-way collinear MAA-based segments were identified with a mean length of 16,426 bp, while only 487 n-way collinear protein-coding genes-based segments were identified; however, as expected, they had a much longer mean length of 124,405 bp (Tables S1, S2 in Supplementary Material). For the 2way-d collinear segments between cucumber and each of the other 14 angiosperm genomes, we identified 80,910 MAAs-based segments with a mean length of 8,934 bp, while there were only 10,632 protein-coding genes-based segments with a mean length of 152,737 bp.

**Figure 4:**
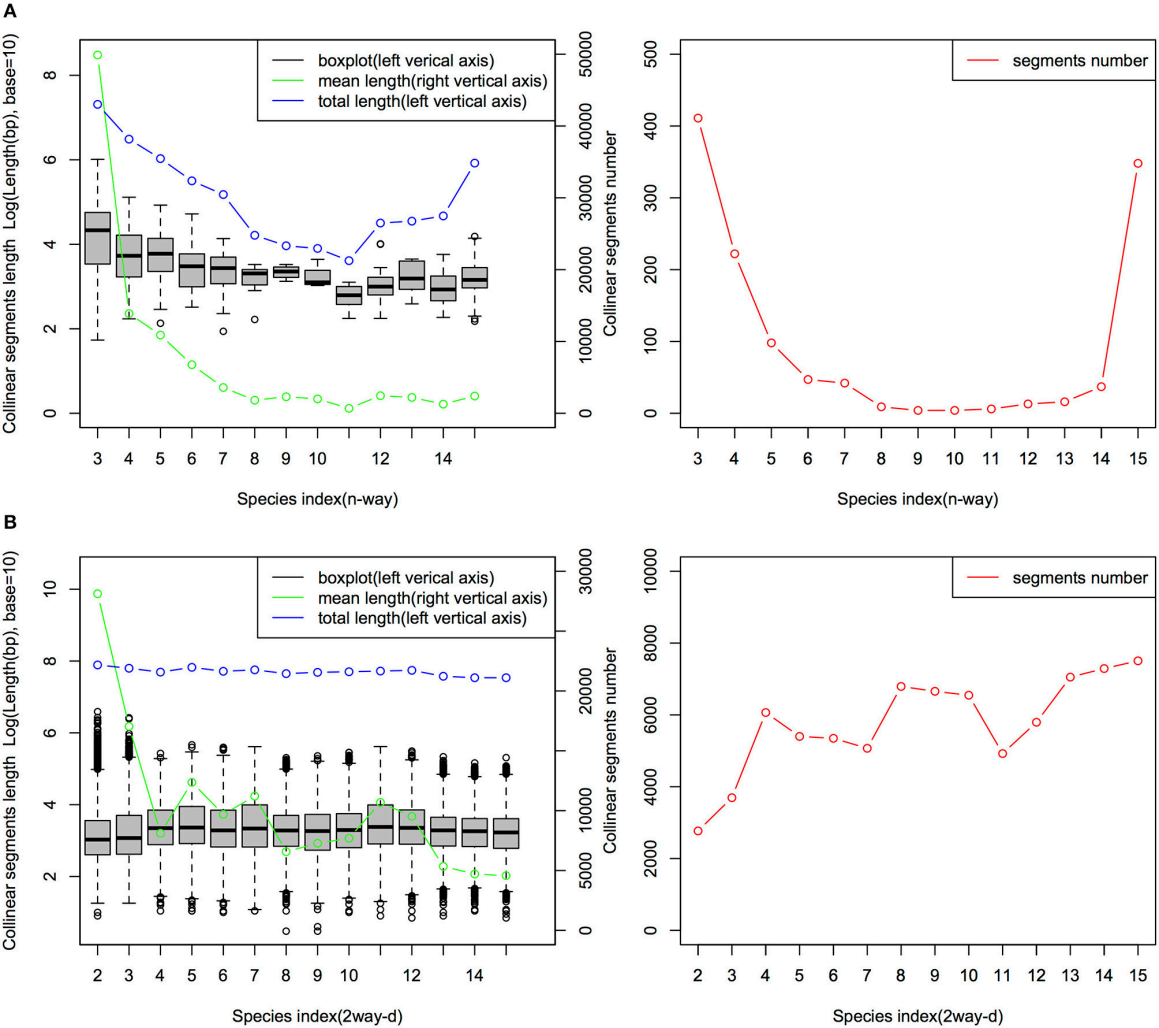
Distribution of n-way **(A)** and 2way-d **(B)** collinear segments (MAAs-based) from 15 angiosperm genomes. The *n*-way (*n*∈ {3,4, …, 15}) collinear segments indicate the group of species consisting of cucumber and other related species which were sequentially incorporated based on the topology of the tree in Figure 1, with cucumber as the origin. These segments represent the multiple species level of collinear segments. Each 2way-*d* (*d*∈ {2,3,…,15}, where *d* is the species index) collinear segment represented a pairwise alignment with one of the 14 non-cucumber species indexed by *d*, where *d* was incremented with the degree of divergence from cucumber according to the phylogenetic tree (Figure 1). Thus, *d* represents, in ascending order, *Cucumis melo, Citrullus lanatus, Malus domestica, Glycine max, Populus trichocarpa, Citrus sinensis, Brassica rapa, Arabidopsis thaliana, Arabidopsis lyrata, Vitis vinifera, Solanum tuberosum, Setaria italica, Brachypodium distachyon, and Oryza brachyantha*.

The characteristics of the collinear MAAs-based segments including total length, segments number and segment length are summarized in Figure 4. The mean length of the n-way collinear MAA-based segments dramatically decreased as the number of included species was increased (Figure 4A), while the distribution of the corresponding segments' total length and segment numbers displayed a U-like shape (Figure 4B). Interestingly, a similar U-like pattern has also been observed in phylogenomic studies of closely related *E. coli* strains (Touchon et al., 2009; Zhang and Lin, 2012). By contrast, the n-way collinear protein-coding genes-based segment number decreased more dramatically with the increase in the number of included species (Figure S5). Importantly, we identified 988 15-way collinear segments by the MAAs-based method, with a mean length of 2,549 bp. Despite the short length of these 15-way segments, they may be valuable for inferring gene orthology and identifying candidate noncoding regulatory elements. Next, Jaccard similarity measures were calculated to compare the collinear MAAs-based segments with segments derived from protein-coding genes. As shown in Table S2 and Figure S6, only a weak relationship was observed at the multiple species level (n-way), although there were pairwise relationships (2way-d) with very high similarity especially between closely related genomes. Though the Jaccard similarity between these two sets of collinear genomic segments decreased rapidly with increasing divergence from cucumber, we found that the MAAs-based method identified a greater number of specific collinear segments than the method based on protein-coding genes. Our results showed that, as one type of potential genome-wide markers, MAAs were more suitable for identifying collinear segments among distantly related genomes than protein-coding genes.

### Characterization of orthologous genes between cucumber and other plants

**Figure 5:**
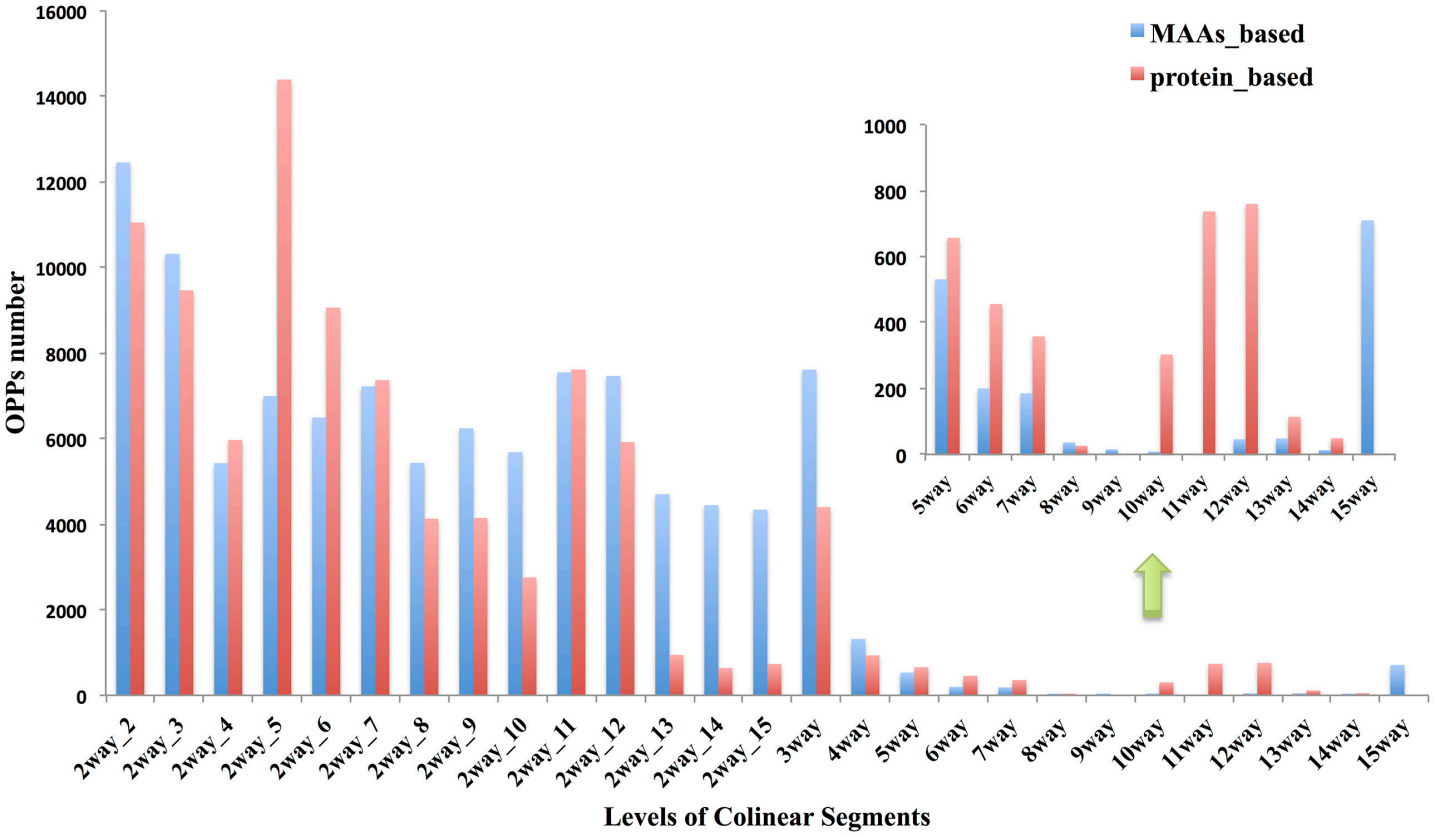
Distribution of OPPs (orthologous protein-coding genes pairs) inferred by different levels of collinear segments. Red bar indicates the results using MAAs as genomic markers; Blue bar is from results using protein-coding genes as markers. The *n*-way(*n*∈ {3,4, …, 15}) collinear segments indicate group of species consisting of cucumber and other related species which were sequentially incorporated based on the topology of the tree in Figure 1, with cucumber as the origin. These segments represent the multiple species level of collinear segments. Each 2way-*d* (*d*∈ {2,3,…,15}, where *d* is the species index) collinear segment represented a pairwise alignment with one of the other 14 non-cucumber species indexed by *d*, where *d* was incremented with the degree of divergence from cucumber according to the phylogenetic tree (Figure 1). Thus, *d* represents, in ascending order, *Cucumis melo, Citrullus lanatus, Malus domestica, Glycine max, Populus trichocarpa, Citrus sinensis, Brassica rapa, Arabidopsis thaliana, Arabidopsis lyrata, Vitis vinifera, Solanum tuberosum, Setaria italica, Brachypodium distachyon*, and *Oryza brachyantha*.

The term “ortholog” originally referred to genes in different species that were derived from the same locus in their last common ancestor. Biologists always, even completely, transferred gene functional information from model species to newly sequenced genomes (Gabaldón and Koonin, 2013) based on the “orthology-function conjecture” rule (Koonin, 2005). This hypothesis stated that orthologs tend to retain similar molecular and biological functions, while paralogs tend to diverge over time to perform different functions via sub- or neo-functionalization. The idea that collinearity among related species may be one of the most reliable methods for orthology inferences has been previously reviewed (Kristensen et al., 2011). Based on the collinear segments identified previously between cucumber and 14 other angiosperms, we inferred 94,486 OPPs according to the orthology-inference rule, as described in the materials and methods. All of the OPPs were shown in Table S11 with MAAs as markers and Table S12 with protein-coding genes as markers. The numerical distribution of the OPPs inferred from different levels of collinear segments was shown in Figure 5.

**Figure 6:**
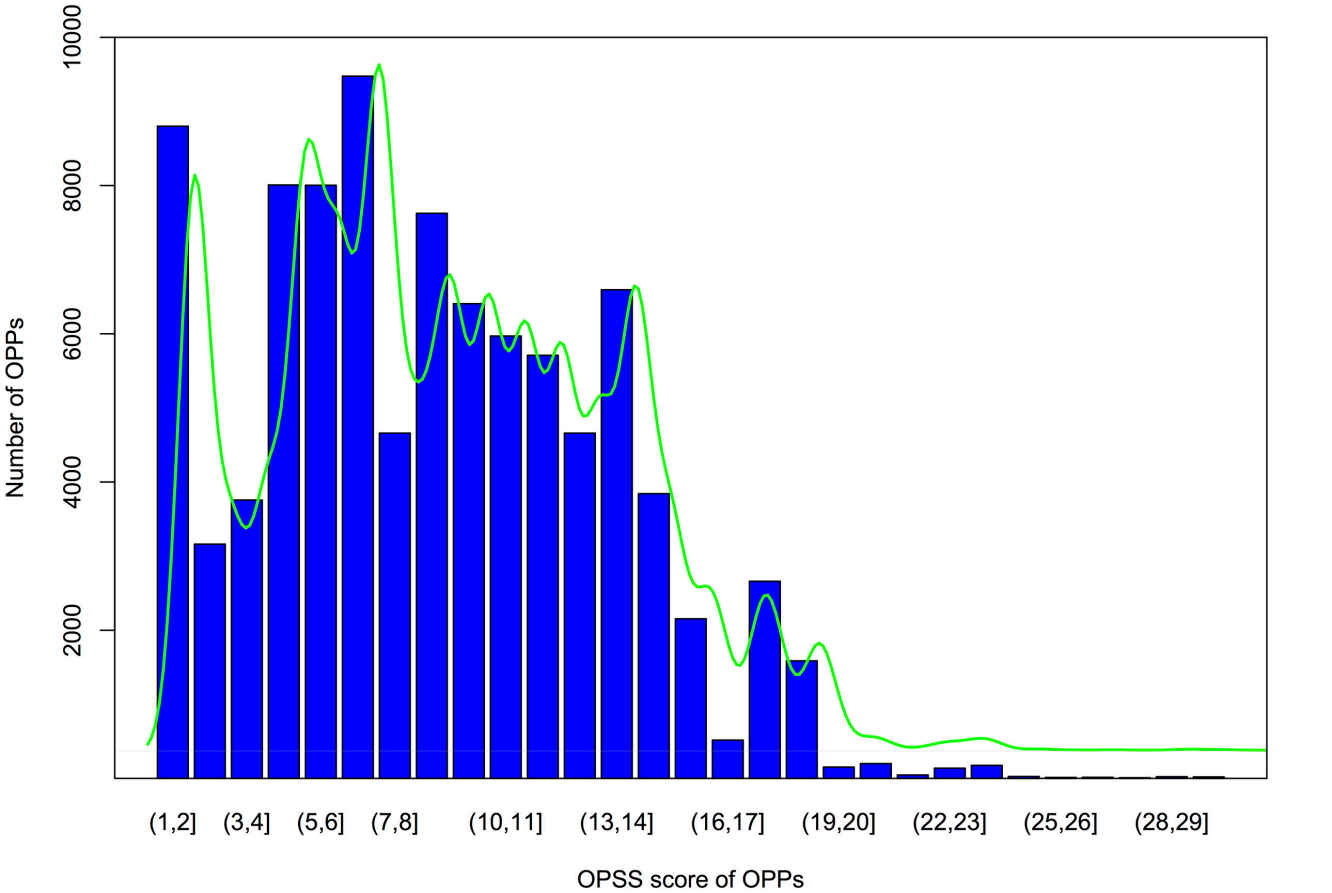
Bar plot of OPSS (orthologous protein-coding genes pair support score) distributions for OPPs (orthologous protein-coding gene pairs). Vertical axis indicates the number of OPPs, and the horizontal ordinate represents OPSS value intervals. The green curve is the fitted density.

Each OPPs was further assigned an OPSS that integrated multiple evidences, including the level of collinearity detected with MAAs or protein-coding genes as genomic markers and sequence similarity (Table S13). Orthologous pair support score calculations were performed as described in the materials and methods. As shown by the OPSS distribution in Figure 6, we found that approximately 75% of OPPs had OPSS values between 5 and 15, indicating that these OPPs were deduced from multiple species level of collinear segments (such as the OPP consisting of Csa1P002120.1 in cucumber and XP_006581876.1 in *G. max*, which was inferred by a 5-way collinear segment) or multiple evidences (such as the OPP consisting of Csa1P050030.1 in cucumber and XP_002864842.1 in *A. thaliana*, which was inferred from a 10-way collinear segment and was also supported by both a 2way-10 collinear segment using protein-coding genes-based method and the BBH strategy). Only 10% of OPPs had OPSS values smaller than 3, indicating that these OPPs were uniquely inferred from a 2way-2 collinear segment identified by the MAAs-based method. Interestingly, 31% of OPPs were validated by the BBH strategy, and 25% of OPPs were deduced from more than one level of collinear segments. For example, the orthologous gene pair Csa1P050470.1 from cucumber and NP_199849.2 from *A. thaliana* was deduced from four types of evidences, including 15-way and 2way-10 collinear segments identified by the MAAs-based method, a 2way-10 collinear segment identified by the protein-coding genes-based method, and the BBH strategy. The distribution of OPPs shown in Figure 6 suggested that the accuracy of the orthology inferences based on collinearity was improved by validation through multiple lines of evidence.

### Functional annotation of predicted protein-coding genes in the cucumber genome

**Figure 7:**
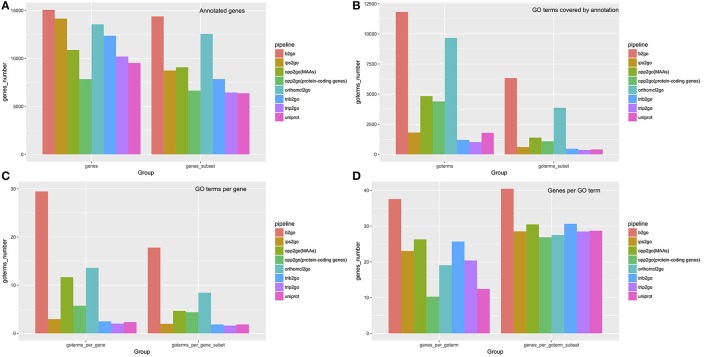
Summary of annotations from 8 pipelines. To compare our annotation results with traditional methods, we selected six widely used pipelines, using default parameters, to assign GO terms to cucumber protein-coding genes, including Blast2GO (abbreviated as b2go), InterProScan (abbreviated as ips2go), OrthoMCL (abbreviated as orthomcl2go), Trinotate-Blast (abbreviated as trib2go), Trinotate-Pfam (abbreviated as trip2go), and the UniProt resource (abbreviated as uniprot). Detailed settings for each pipeline are described in materials and methods. Our pipelines, using MAAs as genomic markers [abbreviated as opp2go (MAAs)] or protein-coding genes [abbreviated as opp2go (protein-coding genes)]. **(A)** Coverage of annotated genes; **(B)** numbers of GO terms covered by annotations; **(C)** average number of GO terms associated with each gene; **(D)** average number of genes annotated by each GO term. “Subset” indicates that GO terms associated with > 300 genes or <5 genes were filtered out.

Guided by the OPPs, we collected functional information of cucumber protein-coding genes whose orthologous genes had been annotated in the UniProt-reference-proteomes database or TAIR (Lamesch et al., 2012). Approximately 44.84% of protein-coding genes (10,885) in the cucumber genome were assigned at least one GO term (Table S4). To reduce noise, we ignored GO terms with >300 or fewer than five associated genes. Overall, each cucumber gene was annotated with an average of 4.66 GO terms, and each GO term was associated with an average of 30.47 genes, as illustrated in Figure 7. In addition, based on the OPPs between cucumber and *A. thaliana*, 4,230 cucumber genes were assigned at least one growth-related Plant Ontology (PO) term (Cooper and Jaiswal, 2016) involving 32 developmental stages or conditions, and 4,797 cucumber genes were annotated with at least one structure-related PO term involving 66 distinct gene expression locations (Table S14).

### Improved functional annotation quality for cucumber protein-coding genes than traditional methods

We compared the annotations derived from a total of eight pipelines, including six traditional methods and our two collinearity-based methods, named opp2go (MAAs-based) and opp2go (protein coding genes-based), with respect to the following aspects: (i) annotated gene coverage and related numbers of GO terms; (ii) the mean number of GO terms per gene and mean number of genes per GO term; and (iii) similarity comparison among these eight annotation results. All the results produced by the eight pipelines were shown in the Tables S4–S10, S15. As illustrated in Figure 7, our opp2go (MAAs-based) pipeline successfully annotated approximately 11,000 protein-coding genes (almost half of the protein-coding genes in cucumber), which was comparable to the results from the 6 traditional methods. By contrast, fewer than 8,000 genes were annotated by the opp2go (protein coding gene-based) method, which was probably due to the weak collinearity identified with protein-coding genes as genomic markers, leading to a smaller number of inferred orthologous genes. Comparing with UniProt resource related with cucumber, the number of genes successfully annotated by opp2go (MAAs-based) was 2,730 more than by UniProt, as shown in Figure 7. Interestingly, there were two valuable observations from the functional annotations in Figure 7. First, the results from our opp2go (MAAs-based) pipeline covered an intermediate number of GO terms, significantly fewer than b2go but more than the two Trinotate-related pipelines and ips2go. In addition, the 4.66 GO terms per gene from opp2go (MAAs-based) was significantly lower than b2go (17.80), but higher than the two Trinotate-related pipelines and ips2go, which had <2 GO terms per gene.

**Figure 8:**
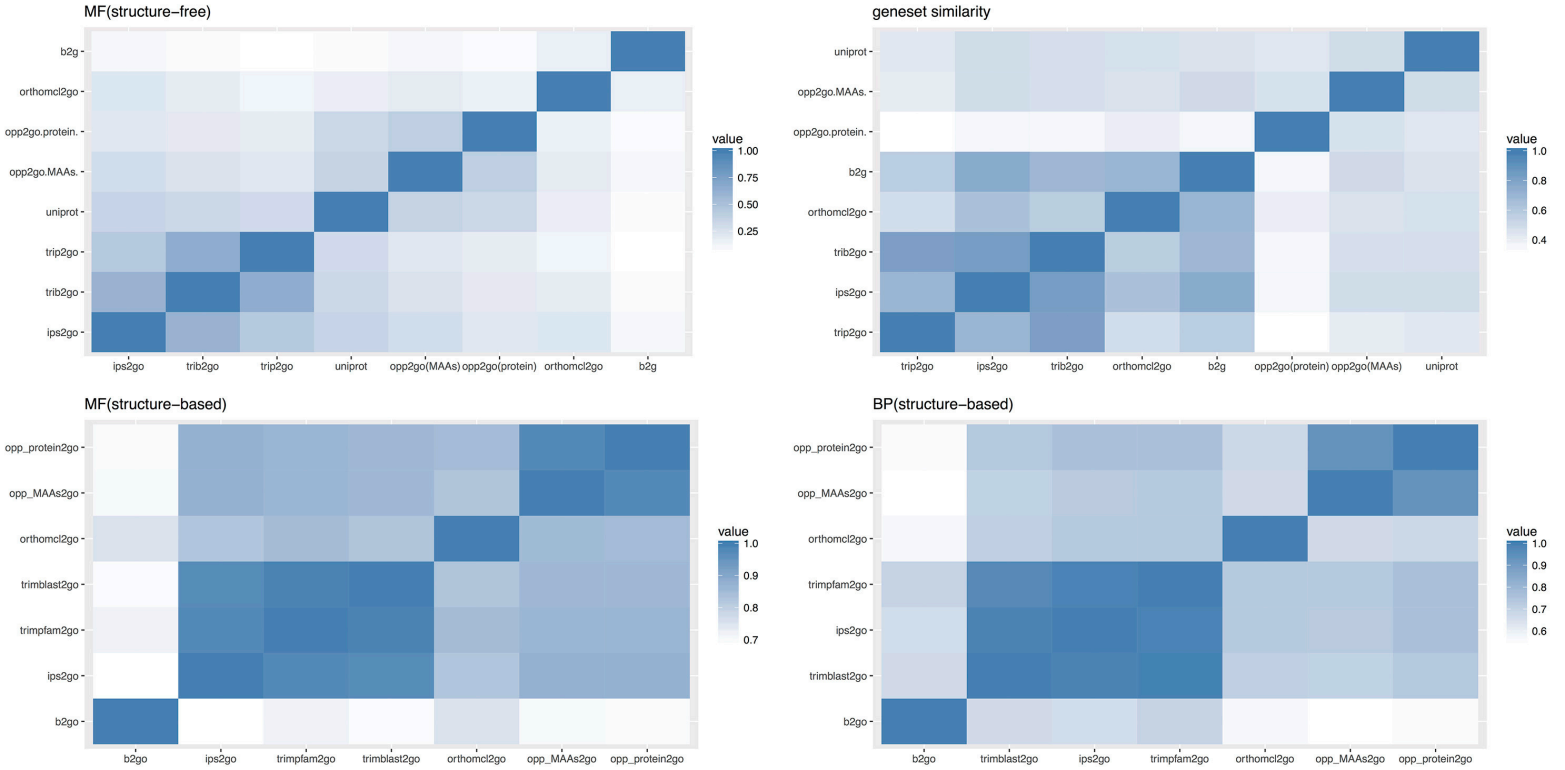
Comparison of annotation results among 8 different pipelines. opp2go (MAAs): our pipeline using MAAs as genomic markers, opp2go (proteins): our pipeline using protein-coding genes as genomic markers, b2go: Blast2GO, ips2go: InterProScan pipeline, orthomcl2go: OrthoMCL pipeline, trib2go: Trinotate-Blast; trip2go: Trinotate-pfam, and uniprot: UniProt resource. MF, molecular function; BP, biological process; CC, cellular component. Top-left indicates the Jaccard similarity for the MF subset (structure-free); Top-right shows annotated gene set similarity (structure-free); Bottom-left indicates the sematic similarity for MF subset (structure-based); Bottom-right indicates the sematic similarity for BP subset (structure-based).

Drawing on the experience of Amar (Amar et al., 2014), we compared these eight pipelines with three types of similarity indices, including gene-GO term pairs, annotated gene sets and overall sematic similarity, as described in the materials and methods. MF (structure-free) of Figure 8 showed the pair-wise Jaccard similarity values for the eight pipelines, measuring the overall similarity between their gene-GO term pairs, using the Molecular Function (MF) subset of GO terms. Though there was a low overall similarity with a mean score of 0.2254, the two collinearity-based pipelines clustered together with UniProt, as did the two Trinotate-related pipelines with ips2go, and b2g clustered with orthomcl2go, which may be partly explained by the similar approaches or shared annotation resources between the members of each cluster. Gene-set similarity of Figure 8 showed the pair-wise similarity values of the gene sets across all of the annotation results, which measured the tendency of any two pipelines to annotate the same genes. Our two collinear-based pipelines and UniProt clustered together and were relatively distant from the other five traditional pipelines. From this, we inferred that collinearity-based pipelines cover different gene sets from traditional methods, which were mainly based on protein sequence similarity. However, even within the same cluster, the gene set similarity between opp2go (MAAs-based) and opp2go (protein-coding genes-based) was only 0.46, indicating that the collinear genomic regions identified with MAA markers were different from that with protein-coding genes as markers. MF and BP (structure-based) in Figure 8 showed the structure-based pair-wise similarity values of gene-GO term pairs among eight pipelines for both MF and Biological Process (BP) GO terms. Structure-based similarity accounts for the parent-children inheritance relationships in the GO hierarchical structure and represents the functional semantic similarity between different pipelines. Overall, the structure-based sematic similarity values were significantly higher than those of structure-free similarity as MF subset in Figure 8, because the structure-based method obtained higher scores with seemingly different but biologically similar predictions. The CC subset also showed similar observation in Figure S7. Our two collinearity-based pipelines formed one cluster with average similarity scores of 0.98 for MF and 0.93 for BP. We also found that the orthomcl2go pipeline (based on gene family information) could be clustered with opp2go (MAAs-based), with sematic similarity scores of 0.83 for MF and 0.67 for BP. By contrast, b2go was the farthest from opp2go (MAAs-based), with similarity scores of 0.70 for MF and 0.55 for BP. Taken together, these observations suggested that, compared with traditional pipelines, our collinearity-based annotation method could annotate protein-coding genes that was comparable to that of b2go and it may capture gene family information from our set of 15 related angiosperm species.

**Figure 9:**
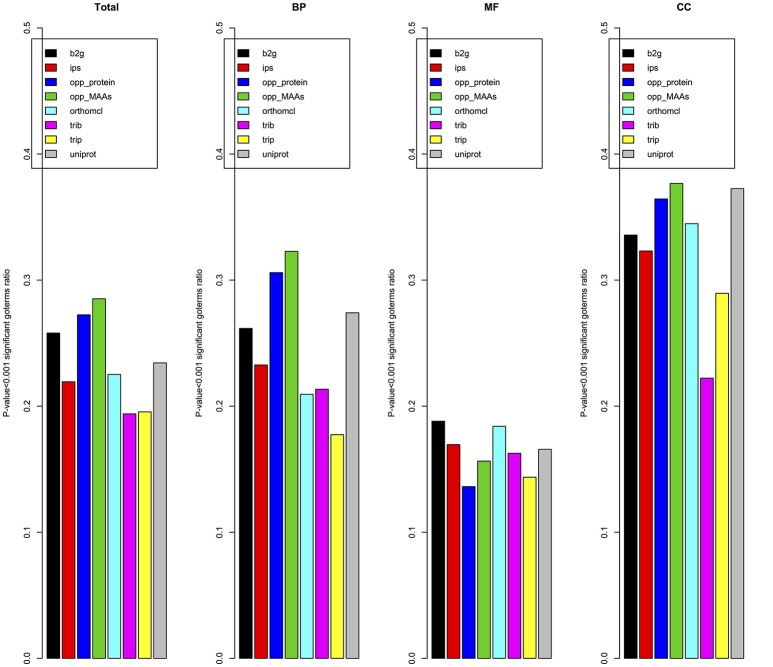
Validation of annotations based on gene co-expression. opp_MAAs, our pipeline using MAAs as genomic markers; opp_protein, our pipeline using protein-coding genes as genomic markers; b2g, Blast2GO; ips, InterProScan pipeline; orthomcl, OrthoMCL pipeline; trib, Trinotate-Blast; trip, Trinotate-pfam, and uniprot, UniProt resource. Given a set of cucumber genes linked to a BP/MF/CC term by a specific pipeline, the average Pearson correlation coefficient for co-expression of genes was compared to that of a random gene set. For each pipeline the number of GO terms with *p*-value < 0.001 was indicated.

Due to the lack of a high-quality gene functional annotation dataset in cucumber, we validated the annotation results using the gene co-expression patterns from expression data of 11,754 genes in 10 different tissues. The Pearson correlation coefficient was used to measure the co-expression between pairs of genes as detailed in materials and methods. To reduce the GO annotation bias, we removed GO terms associated with more than 300 genes or fewer than 5 genes. Figure 9 showed the ratio of specific GO terms with *P*-values < 0.001 for each pipeline. The opp2go method (MAAs-based) showed the best performance among the eight pipelines for the total GO terms and both the BP and Cellular Component (CC) GO term subsets. However, for the MF subset of GO terms, the ratios of significantly overrepresented terms (*P*-value < 0.001) among opp2go (MAA-based) and the other methods were all <0.2. We also observed that all eight pipelines showed better performance for the CC subset than BP and MF. One explanation might be that co-expression between genes was evaluated using RNA expression datasets from 10 specific tissues, in which they might contain some degree of tissue-specific and cellular component information. In summary, the annotation quality of opp2go (MAA-based) obtaineds a partial improvement over those of traditional methods, which might be due to its integration of collinearity between related species into the functional annotation process. Our results also demonstrated that inter-species collinearity was a reliable basis for orthologous gene inference.

## Discussion

Using a LASTZ/MULTIZ analysis pipeline, we identified a class of DNA segments (MAAs) that was highly conserved across 15 angiosperm genomes. These MAAs were more numerous, shorter in length, more widely and uniformly distributed than protein-coding genes in cucumber. Using MAAs as genomic markers, we identified multiple levels of collinear segments between cucumber and 14 related species. According to our survey, the mean length of collinear segments decreased dramatically with increasing genetic divergence from cucumber. However, with increasing divergence from cucumber, the number of segments gradually increased in the MAAs-based results, while a nearly opposite trend was observed with protein-coding genes. As a possible explanation, we hypothesized that long collinear segments might have been split into multiple smaller segments due to genomic events that disturbed these plant genomic segments over the course of long-term evolution. In this study, no 9- or 15-way collinear segments were identified by the protein-coding genes-based method, which suggests that using MAAs as genomic markers may be a viable alternative to protein-coding genes for detecting collinear segments among distantly related plants. More importantly, due to the co-localization of genes and their respective regulatory elements, the collinear segments may be used to detect potential *cis*-regulatory elements for nearby target genes as reviewed previously (Levy et al., 2001; Wittkopp and Kalay, 2011).

With these identified collinear segments, we inferred putative OPPs between cucumber and each of the other 14 species. Each of these orthologs to cucumber genes was then used as a proxy to transfer annotation information to its corresponding cucumber gene if its biological function was known. Although the annotation coverage was comparable with traditional methods, our functional annotation strategy greatly reduced the annotation redundancy and obtained a partial improvement of annotation accuracy evaluated by gene co-expression profiles.

However, the criteria for MAA inclusion should be refined by additional steps, including phylogenetic and evolutionary model analyses, such as BinCons (Margulies et al., 2003) or GERP (Cooper et al., 2005), sequence characters and lineage-specific information. Additionally, it is important to consider that genes tend to diverge over time to perform different functions at the domain level, rather than across the whole gene (Rentzsch and Orengo, 2013). In the future, we plan to improve our annotation pipeline by performing functional annotation of protein-coding genes based on domain level. The comparison of plant genomes has been complicated by recurrent polyploidy and extensive genome rearrangements (Tang et al., 2008), which strongly impact genome alignment and the identification of collinear segments. So, our current results should be interpreted cautiously until these inferred collinear segments have been supported by additional lines of evidence.

In summary, using the cucumber as a case study, we provided a potential alternative resource for the functional annotation of cucumber protein-coding genes that was an alternative strategy for transferring functional information from previously well-characterized protein-coding genes in model species to genes in “non-model” plant species guided by genomic collinearity, accessible from http://cmb.bnu.edu.cn/functional_annotation. In addition, our study offers a pipeline for identifying collinear segments across multiple related plant genomes based on conserved DNA segments, which can potentially be used as genomic markers instead of protein-coding genes.

## Author contributions

EP and KL: contributed the central idea; HS and EP: designed the study; HS: analyzed most of the data and wrote the initial draft of the paper; JH: contributed to refining the ideas. All authors discussed the results and revised the manuscript.

### Conflict of interest statement

The authors declare that the research was conducted in the absence of any commercial or financial relationships that could be construed as a potential conflict of interest.

## Abbreviations

MAAs: Multiple alignment anchors
OPPs: Orthology protein-coding gene pairs
OPSS: orthology pair support score.

## References

[B1] Amar D. Frades I. Danek A. Goldberg T. Sharma S. K. Hedley P. E.. (2014). Evaluation and integration of functional annotation pipelines for newly sequenced organisms: the potato genome as a test case. BMC Plant Biol. 14:329. 10.1186/s12870-014-0329-9 25476999 PMC4274702

[B2] Bennetzen J. L. Schmutz J. Wang H. Percifield R. Hawkins J. Pontaroli A. C.. (2012). Reference genome sequence of the model plant Setaria. Nat. Biotechnol. 30, 555–561. 10.1038/nbt.2196 22580951

[B3] Blanchette M. Kent W. J. Riemer C. Elnitski L. Smit A. F. Roskin K. M.. (2004). Aligning multiple genomic sequences with the threaded blockset aligner. Genome Res. 14, 708–715. 10.1101/gr.1933104 15060014 PMC383317

[B4] Bowers J. E. Chapman B. A. Rong J. Paterson A. H. (2003). Unravelling angiosperm genome evolution by phylogenetic analysis of chromosomal duplication events. Nature 422, 433–438. 10.1038/nature01521 12660784

[B5] Burge S. Kelly E. Lonsdale D. Mutowo-Muellenet P. McAnulla C. Mitchell A.. (2012). Manual GO annotation of predictive protein signatures: the InterPro approach to GO curation. Database 2012:bar068. 10.1093/database/bar068 22301074 PMC3270475

[B6] Chen J. Huang Q. Gao D. Wang J. Lang Y. Liu T.. (2013). Whole-genome sequencing of *Oryza brachyantha* reveals mechanisms underlying Oryza genome evolution. Nat. Commun. 4:1595. 10.1038/ncomms2596 23481403 PMC3615480

[B7] Conesa A. Götz S. García-Gómez J. M. Terol J. Talón M. Robles M. (2005). Blast2GO: a universal tool for annotation, visualization and analysis in functional genomics research. Bioinformatics 21, 3674–3676. 10.1093/bioinformatics/bti610 16081474

[B8] Cooper G. M. Stone E. A. Asimenos G. Program N. C. S. Green E. D. Batzoglou S.. (2005). Distribution and intensity of constraint in mammalian genomic sequence. Genome Res. 15, 901–913. 10.1101/gr.3577405 15965027 PMC1172034

[B9] Cooper L. Jaiswal P. (2016). The plant ontology: a tool for plant genomics. Methods Mol. Biol. 1374, 89–114. 10.1007/978-1-4939-3167-5_5 26519402

[B10] Davies T. J. Barraclough T. G. Chase M. W. Soltis P. S. Soltis D. E. Savolainen V. (2004). Darwin's abominable mystery: insights from a supertree of the angiosperms. Proc. Natl. Acad. Sci. U.S.A. 101, 1904–1909. 10.1073/pnas.0308127100 14766971 PMC357025

[B11] Gabaldón T. Koonin E. V. (2013). Functional and evolutionary implications of gene orthology. Nat. Rev. Genet. 14, 360–366. 10.1038/nrg3456 23552219 PMC5877793

[B12] Garcia-Mas J. Benjak A. Sanseverino W. Bourgeois M. Mir G. González V. M.. (2012). The genome of melon (*Cucumis melo* L.). Proc. Natl. Acad. Sci. U.S.A. 109, 11872–11877. 10.1073/pnas.1205415109 22753475 PMC3406823

[B13] Grabherr M. G. Haas B. J. Yassour M. Levin J. Z. Thompson D. A. Amit I.. (2011). Full-length transcriptome assembly from RNA-Seq data without a reference genome. Nat. Biotechnol. 29, 644–652. 10.1038/nbt.1883 21572440 PMC3571712

[B14] Guo S. Zhang J. Sun H. Salse J. Lucas W. J. Zhang H.. (2013). The draft genome of watermelon (*Citrullus lanatus*) and resequencing of 20 diverse accessions. Nat. Genet. 45, 51–58. 10.1038/ng.2470 23179023

[B15] Harris R. S. (2007). Improved Pairwise Alignment of Genomic DNA. Ph.D. Thesis, The Pennsylvania State University.

[B16] Hu T. T. Pattyn P. Bakker E. G. Cao J. Cheng J. F. Clark R. M.. (2011). The *Arabidopsis lyrata* genome sequence and the basis of rapid genome size change. Nat. Genet. 43, 476–481. 10.1038/ng.807 21478890 PMC3083492

[B17] Huang S. Li R. Zhang Z. Li L. Gu X. Fan W.. (2009). The genome of the cucumber, *Cucumis sativus* L. Nat. Genet. 41, 1275–1281. 10.1038/ng.475 19881527

[B18] Hupalo D. Kern A. D. (2013). Conservation and functional element discovery in 20 angiosperm plant genomes. Mol. Biol. Evol. 30, 1729–1744. 10.1093/molbev/mst082 23640124

[B19] International Brachypodium I. (2010). Genome sequencing and analysis of the model grass *Brachypodium distachyon*. Nature 463, 763–768. 10.1038/nature08747 20148030

[B20] Jaccard P. (1912). The distribution of the flora in the alpine Zone.1. New Phytol. 11, 37–50. 10.1111/j.1469-8137.1912.tb05611.x

[B21] Jaillon O. Aury J. M. Noel B. Policriti A. Clepet C. Casagrande A.. (2007). The grapevine genome sequence suggests ancestral hexaploidization in major angiosperm phyla. Nature 449, 463–467. 10.1038/nature06148 17721507

[B22] Jansen A. Verstrepen K. J. (2011). Nucleosome positioning in *Saccharomyces cerevisiae*. Microbiol. Mol. Biol. Rev. 75, 301–320. 10.1128/MMBR.00046-10 21646431 PMC3122627

[B23] Jones P. Binns D. Chang H. Y. Fraser M. Li W. McAnulla C.. (2014). InterProScan 5: genome-scale protein function classification. Bioinformatics 30, 1236–1240. 10.1093/bioinformatics/btu031 24451626 PMC3998142

[B24] Kent W. J. Baertsch R. Hinrichs A. Miller W. Haussler D. (2003). Evolution's cauldron: duplication, deletion, and rearrangement in the mouse and human genomes. Proc. Natl. Acad. Sci. U.S.A. 100, 11484–11489. 10.1073/pnas.1932072100 14500911 PMC208784

[B25] Koonin E. V. (2005). Orthologs, paralogs, and evolutionary genomics. Annu. Rev. Genet. 39, 309–338. 10.1146/annurev.genet.39.073003.114725 16285863

[B26] Kristensen D. M. Wolf Y. I. Mushegian A. R. Koonin E. V. (2011). Computational methods for gene orthology inference. Brief. Bioinformatics 12, 379–391. 10.1093/bib/bbr030 21690100 PMC3178053

[B27] Lamesch P. Berardini T. Z. Li D. Swarbreck D. Wilks C. Sasidharan R.. (2012). The Arabidopsis Information Resource (TAIR): improved gene annotation and new tools. Nucleic Acids Res. 40, D1202–D1210. 10.1093/nar/gkr1090 22140109 PMC3245047

[B28] Levy S. Hannenhalli S. Workman C. (2001). Enrichment of regulatory signals in conserved non-coding genomic sequence. Bioinformatics 17, 871–877. 10.1093/bioinformatics/17.10.871 11673231

[B29] Li L. Stoeckert C. J. Jr. Roos D. S. (2003). OrthoMCL: identification of ortholog groups for eukaryotic genomes. Genome Res. 13, 2178–2189. 10.1101/gr.1224503 12952885 PMC403725

[B30] Li Z. Zhang Z. Yan P. Huang S. Fei Z. Lin K. (2011). RNA-Seq improves annotation of protein-coding genes in the cucumber genome. BMC Genomics 12:540. 10.1186/1471-2164-12-540 22047402 PMC3219749

[B31] Margulies E. H. Blanchette M. Program N. C. S. Haussler D. Green E. D. (2003). Identification and characterization of multi-species conserved sequences. Genome Res. 13, 2507–2518. 10.1101/gr.1602203 14656959 PMC403793

[B32] Nehrt N. L. Clark W. T. Radivojac P. Hahn M. W. (2011). Testing the ortholog conjecture with comparative functional genomic data from mammals. PLoS Comput. Biol. 7:e1002073. 10.1371/journal.pcbi.1002073 21695233 PMC3111532

[B33] Potato Genome Sequencing C. Xu X. Pan S. Cheng S. Zhang B. Mu D. (2011). Genome sequence and analysis of the tuber crop potato. Nature 475, 189–195. 10.1038/nature10158 21743474

[B34] Proost S. Fostier J. De Witte D. Dhoedt B. Demeester P. Van de Peer Y.. (2012). i-ADHoRe 3.0–fast and sensitive detection of genomic homology in extremely large data sets. Nucleic Acids Res. 40:e11. 10.1093/nar/gkr955 22102584 PMC3258164

[B35] Rentzsch R. Orengo C. A. (2013). Protein function prediction using domain families. BMC Bioinformatics 14(Suppl. 3), S5. 10.1186/1471-2105-14-S3-S5 23514456 PMC3584934

[B36] Schmutz J. Cannon S. B. Schlueter J. Ma J. Mitros T. Nelson W.. (2010). Genome sequence of the palaeopolyploid soybean. Nature 463, 178–183. 10.1038/nature08670 20075913

[B37] Shang Y. Ma Y. Zhou Y. Zhang H. Duan L. Chen H.. (2014). Plant science. Biosynthesis, regulation, and domestication of bitterness in cucumber. Science 346, 1084–1088. 10.1126/science.1259215 25430763

[B38] Siepel A. Bejerano G. Pedersen J. S. Hinrichs A. S. Hou M. Rosenbloom K.. (2005). Evolutionarily conserved elements in vertebrate, insect, worm, and yeast genomes. Genome Res. 15, 1034–1050. 10.1101/gr.3715005 16024819 PMC1182216

[B39] Swarbreck D. Wilks C. Lamesch P. Berardini T. Z. Garcia-Hernandez M. Foerster H.. (2008). The Arabidopsis Information Resource (TAIR): gene structure and function annotation. Nucleic Acids Res. 36, D1009–D1014. 10.1093/nar/gkm965 17986450 PMC2238962

[B40] Tang H. Bowers J. E. Wang X. Ming R. Alam M. Paterson A. H. (2008). Synteny and collinearity in plant genomes. Science 320, 486–488. 10.1126/science.1153917 18436778

[B41] Tanurdzic M. Banks J. A. (2004). Sex-determining mechanisms in land plants. Plant Cell 16(Suppl.), S61–S71. 10.1105/tpc.016667 15084718 PMC2643385

[B42] Touchon M. Hoede C. Tenaillon O. Barbe V. Baeriswyl S. Bidet P.. (2009). Organised genome dynamics in the *Escherichia coli* species results in highly diverse adaptive paths. PLoS Genet. 5:e1000344. 10.1371/journal.pgen.1000344 19165319 PMC2617782

[B43] Trapnell C. Pachter L. Salzberg S. L. (2009). TopHat: discovering splice junctions with RNA-Seq. Bioinformatics 25, 1105–1111. 10.1093/bioinformatics/btp120 19289445 PMC2672628

[B44] Trapnell C. Williams B. A. Pertea G. Mortazavi A. Kwan G. van Baren M. J.. (2010). Transcript assembly and quantification by RNA-Seq reveals unannotated transcripts and isoform switching during cell differentiation. Nat. Biotechnol. 28, 511–515. 10.1038/nbt.1621 20436464 PMC3146043

[B45] Tuskan G. A. Difazio S. Jansson S. Bohlmann J. Grigoriev I. Hellsten U.. (2006). The genome of black cottonwood, *Populus trichocarpa* (Torr. & Gray). Science 313, 1596–1604. 10.1126/science.1128691 16973872

[B46] Velasco R. Zharkikh A. Affourtit J. Dhingra A. Cestaro A. Kalyanaraman A.. (2010). The genome of the domesticated apple (*Malus x domestica* Borkh.). Nat. Genet. 42, 833–839. 10.1038/ng.654 20802477

[B47] Wang J. Z. Du Z. Payattakool R. Yu P. S. Chen C. F. (2007). A new method to measure the semantic similarity of GO terms. Bioinformatics 23, 1274–1281. 10.1093/bioinformatics/btm087 17344234

[B48] Wang X. Wang H. Wang J. Sun R. Wu J. Liu S.. (2011). The genome of the mesopolyploid crop species *Brassica rapa*. Nat. Genet. 43, 1035–1039. 10.1038/ng.919 21873998

[B49] Wittkopp P. J. Kalay G. (2011). Cis-regulatory elements: molecular mechanisms and evolutionary processes underlying divergence. Nat. Rev. Genet. 13, 59–69. 10.1038/nrg3095 22143240

[B50] Xu Q. Chen L. L. Ruan X. Chen D. Zhu A. Chen C.. (2013). The draft genome of sweet orange (*Citrus sinensis*). Nat. Genet. 45, 59–66. 10.1038/ng.2472 23179022

[B51] Zhang Y. Lin K. (2012). A phylogenomic analysis of *Escherichia coli* / Shigella group: implications of genomic features associated with pathogenicity and ecological adaptation. BMC Evol. Biol. 12:174 10.1186/1471-2148-12-174 22958895 PMC3444427

[B52] Zheng X. H. Lu F. Wang Z. Y. Hoover J. Mural R. (2005). Using shared genomic synteny and shared protein functions to enhance the identification of orthologous gene pairs. Bioinformatics 21, 703–710. 10.1093/bioinformatics/bti045 15458983

