# Supplementary figures and images for: An Updated Functional Annotation of Protein-Coding Genes in the Cucumber Genome
Source: Front Plant Sci. 2018 Mar 15;9:325. doi: 10.3389/fpls.2018.00325 (PMC5863696; doi:10.3389/fpls.2018.00325)

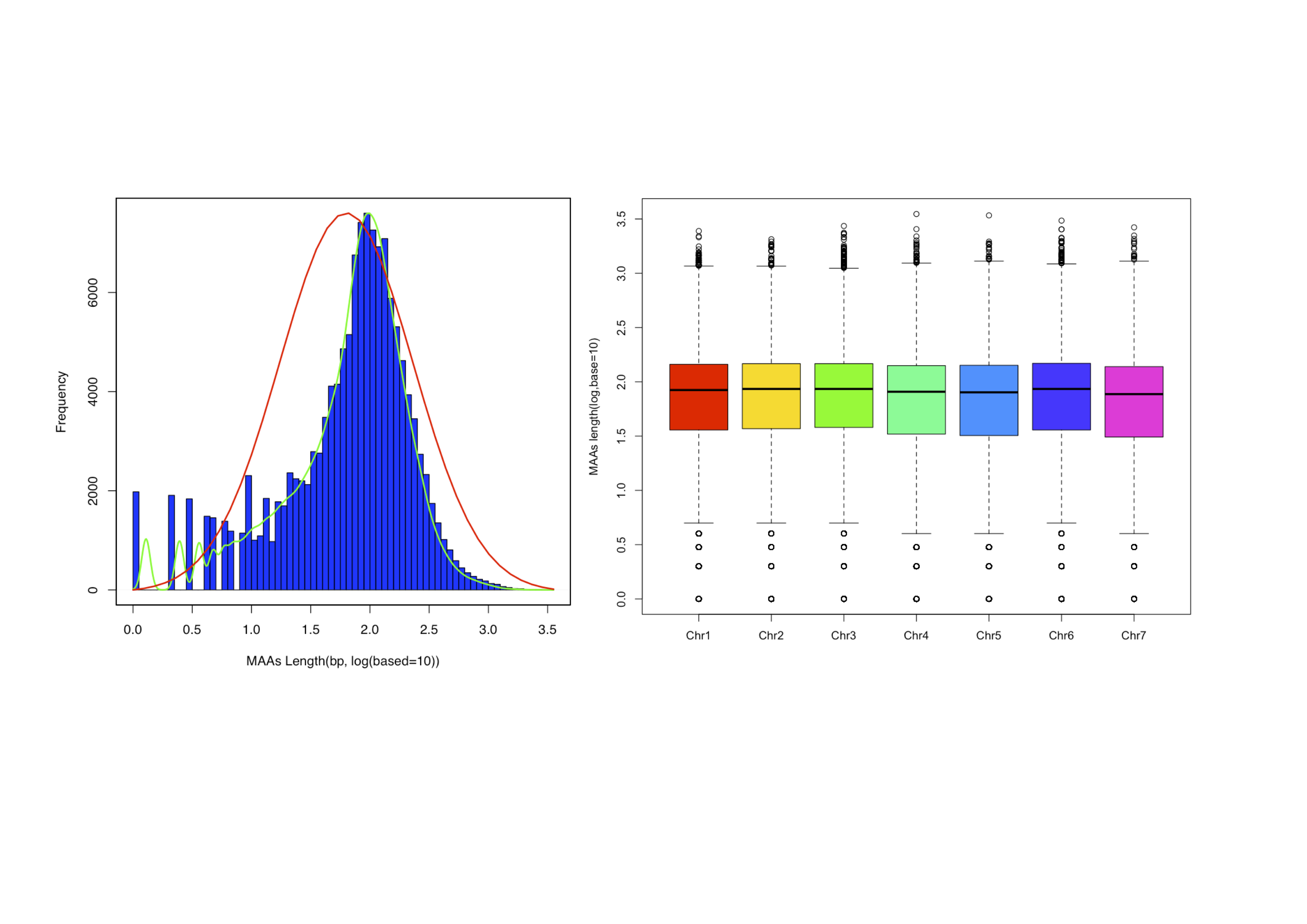

Supplement: Supplementary file 1 [file Image1.TIFF]

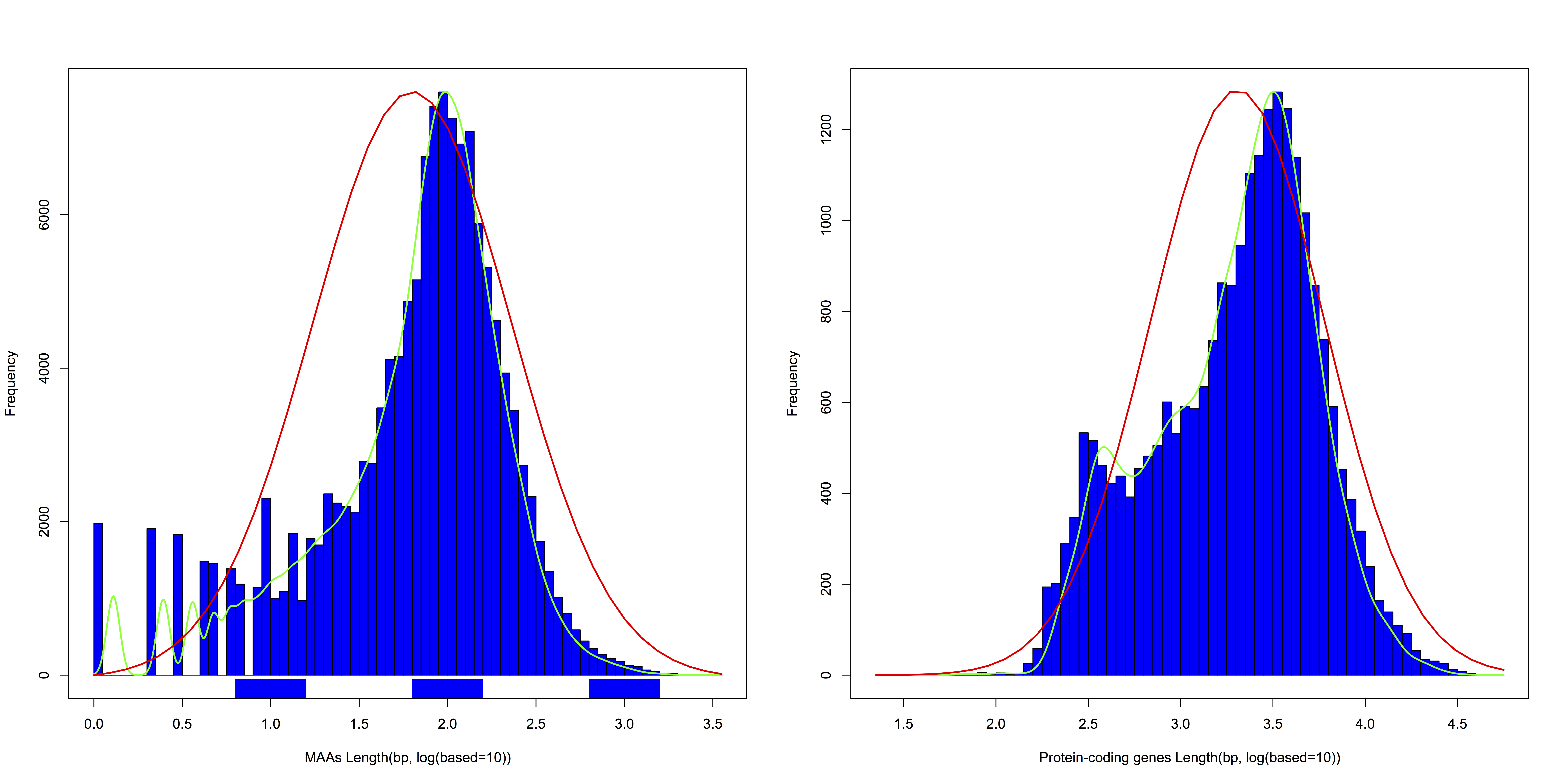

Supplement: Supplementary file 2 [file Image2.TIFF]

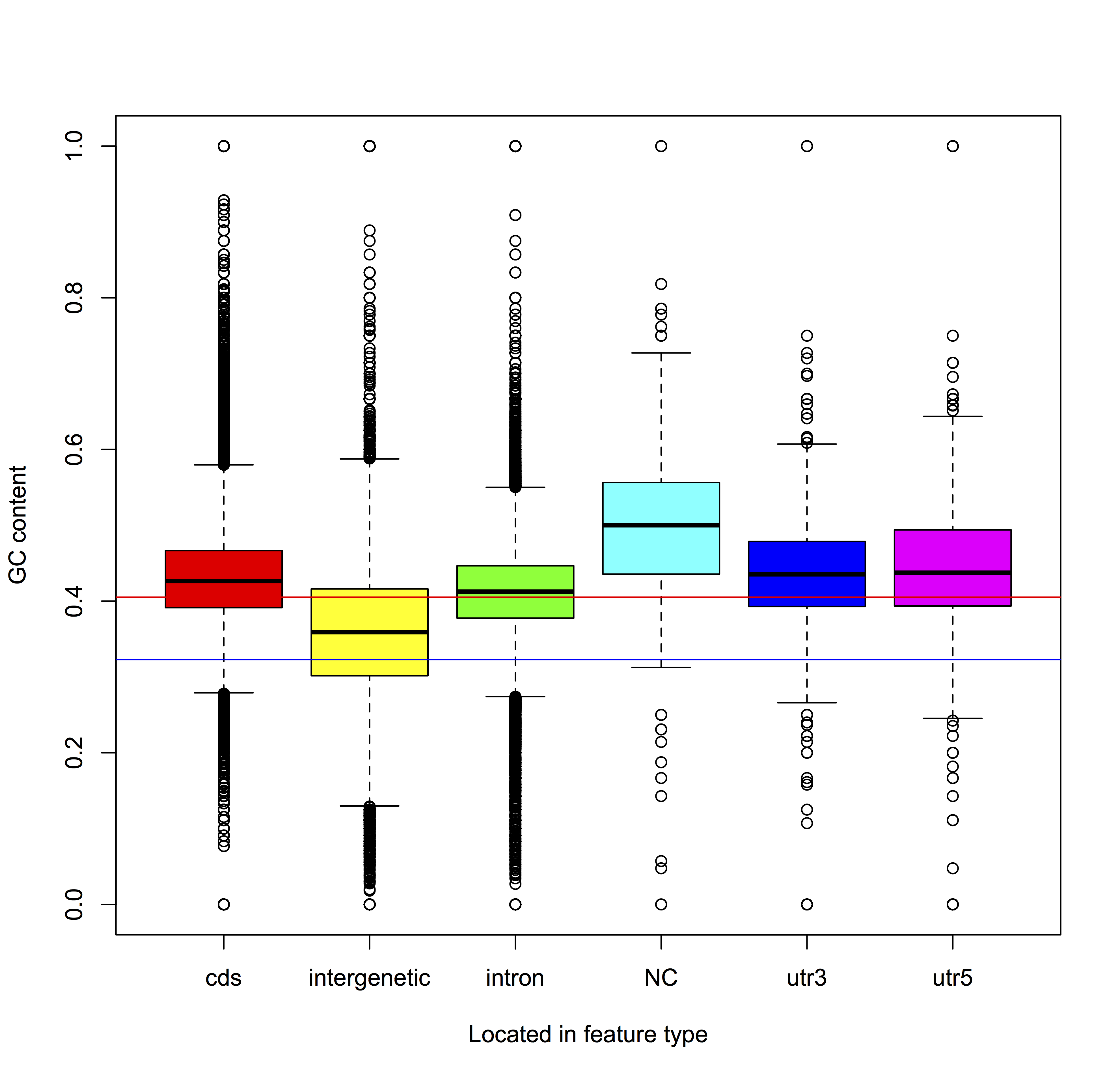

Supplement: Supplementary file 3 [file Image3.TIFF]

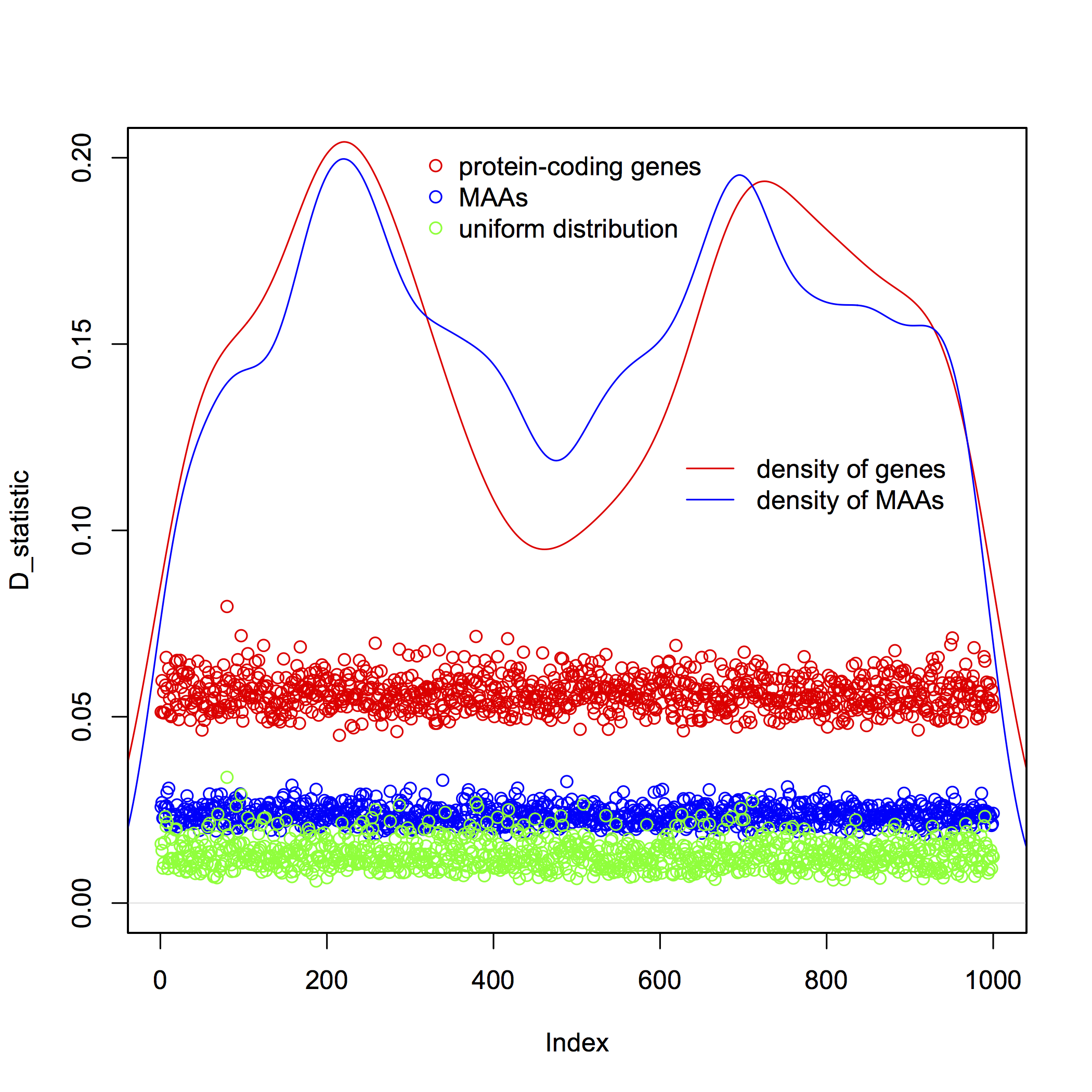

Supplement: Supplementary file 4 [file Image4.TIFF]

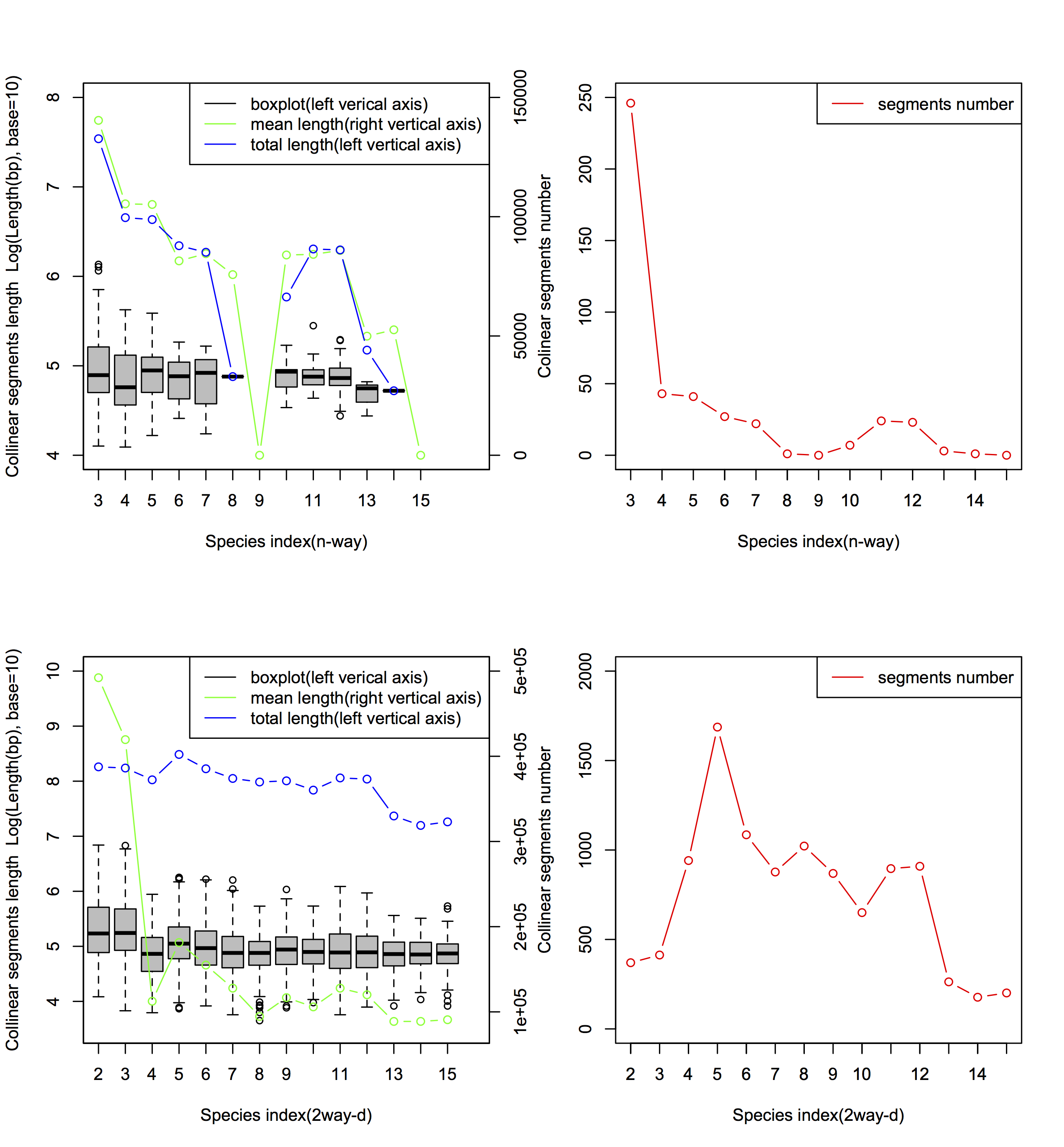

Supplement: Supplementary file 5 [file Image5.TIFF]

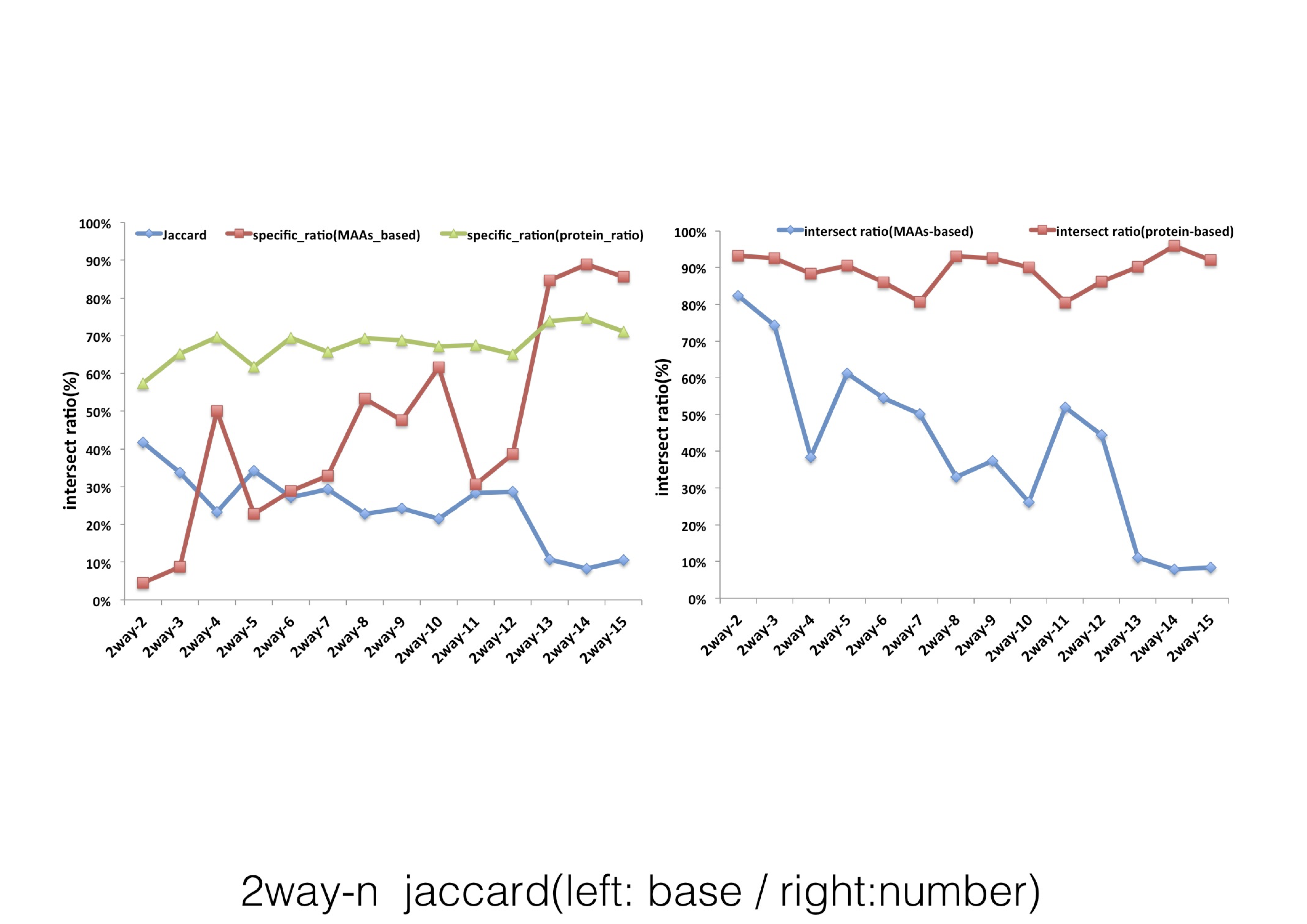

Supplement: Supplementary file 6 [file Image6.TIFF]

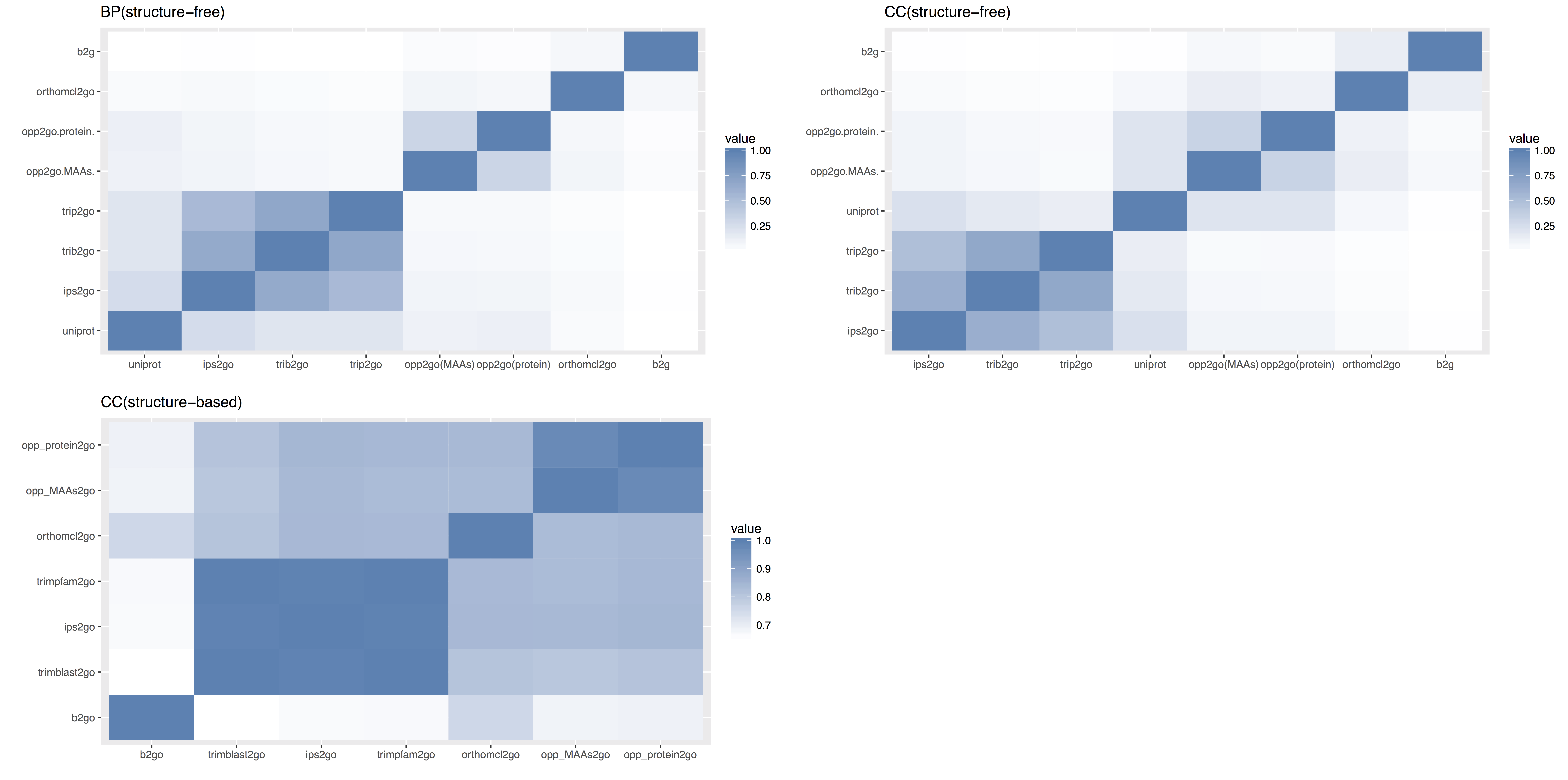

Supplement: Supplementary file 7 [file Image7.TIFF]
