# Supplementary material for: An Updated Functional Annotation of Protein-Coding Genes in the Cucumber Genome
Source: Front Plant Sci. 2018 Mar 15;9:325. doi: 10.3389/fpls.2018.00325 (PMC5863696; doi:10.3389/fpls.2018.00325)
Supplement: Supplementary file 21 [file Presentation1.PDF]

Supplementary Materials

An updated functional annotation of protein-coding genes in the cucumber genome

Hongtao Song[1], Kui Lin[1], Jinglu Hu[2], Erli Pang[1*]

[1]MOE Key Laboratory for Biodiversity Science and Ecological Engineering, College of Life Sciences, Beijing Normal University, Beijing, China

[2]Graduate School of Information, Production and Systems, Waseda University, Wakamatsu-ku, Kitakyushu-shi, Fukuoka-ken, Japan

*** Correspondence:**
Erli Pang
pangerli@bnu.edu.cn

1 Other six commonly-used pipelines for gene functional annotation

In order to evaluate the functional annotation quality of our collinearity-based pipeline, we chose 6 other common-used annotation pipelines, including Blast2GO(Conesa et al., 2005), OrthoMCL(Li et al., 2003), InterPro2GO(Jones et al., 2014), Trinotate-Blast, Trinotate-pfam(Grabherr et al., 2011) and UniProt resource. Detailed pipelines settings and procedures as follows:

1) Blast2GO pipeline: The total protein sequences from cucumber genome were blasted by NCBI-Blast-2.2.26 (BlastP, E-value=1E-05) against SwissProt database (release_2013-05). The blastp outputs were loaded into Blast2GO(Conesa et al., 2005) with default settings and then it assigned GO terms for each of protein-coding genes in cucumber.

2) OrthoMCL pipeline: First, we constructed the protein-coding genes family among 15 angiosperms plants by the OrthoMCL(Li et al., 2003). Second, the total protein sequences from 15 angiosperms plants were blasted by NCBI-Blast-2.2.26 against UniProt database (release_2013-07) with an E-value cutoff of 1E-05. For each protein sequence we kept a ranked list of ten best hits. The first hit in the list that had GO annotation in UniProt was associated with the protein. An OrthoMCL cluster group inherited all GO terms associated with its proteins, and each cucumber protein-coding gene inherited the GO terms of all its group members.

3) InterProScan pipeline: The protein sequences of cucumber were scanned by InterProScan-5.3-46.0(Jones et al., 2014)(default settings, -goterms). The GO terms assigned to all domains of each cucumber protein-coding gene were collected and gathered together as the gene's final functional annotation.

4) Trinotate pipeline: According to the annotation resource database, it could be divided into two Trinotate-related methods. Trinotate-Blast (trimblast2go) relied on the results of Blast against the SwissProt database (release_2013_05), and Trinotate-pfam (trimpfam2go) used HMMER(Finn et al., 2015) to search the Pfam-A database (Finn et al., 2014). Guided by the online steps (http://trinotate.github.io), trinotate (Grabherr et al., 2011) could collect the functional annotations (GO terms) for each cucumber protein-coding gene from SwissProt or Pfam databases.

5) UniProt resource: we downloaded Cucumber UniProt-reference-proteomes from ftp://ftp.uniprot.org/pub/databases/uniprot/current_release/knowledgebase/reference_proteomes and associated all the go-terms recorded in UniProt-reference-proteomes annotation with its protein-coding genes in cucumber.

2 Supplementary tables

Table S1 Similarity information of n-way collinear segments by two methods (MAAs-based and protein-based) with base level*

n-way	Total length (M)	Total length (P)	Merged length	Intersect length	Jaccard index	specific_ratio (M)	specific_ratio (P)
3-way	19.470.904	33.895.249	48.583.5	4.782.643	9.84%	75.44%	85.89%
4-way	2,876,541	3,510,284	6,215,68	171,137	2.75%	94.05%	95.12%
5-way	984,117	2,341,853	3,190,17	135,800	4.26%	86.20%	94.20%
6-way	260,332	1,383,827	1,640,51	3,645	0.22%	98.60%	99.74%
7-way	149,410	789,810	939,220	0	0.00%	100.00%	100.00%
8-way	16,351	75,725	92,076	0	0.00%	100.00%	100.00%
9-way	9,219	0	9,219	0	0.00%	100.00%	0.00%
10-way	7,980	426,635	434,615	0	0.00%	100.00%	100.00%
11-way	4,096	1,232,414	1,236,51	0	0.00%	100.00%	100.00%
12-way	31,847	1,209,466	1,241,31	0	0.00%	100.00%	100.00%
13-way	28,797	132,743	161,540	0	0.00%	100.00%	100.00%
14-way	39,268	52,587	91,855	0	0.00%	100.00%	100.00%
15-way	366,249	0	366,249	0	0.00%	100.00%	0.00%

*base level: with base-pair as the unit. M indicated the results using MAAs as markers, and P indicated that of using protein-coding genes as markers. NA indicated that no segment was identified by the method as denominator. ‘specific’ indicated that the segments were only identified by the method.

Table S2 Similarity information of n-way collinear segments by two methods (MAAs-based and protein-based) with segment level*

n-way	Total num. (M)	Total num. (P)	Specific num. (M)	Specific num. (P)	Intersect num. (M by P)	Intersect num. (P by M)	Intersect ratio (M)	Intersect ratio (P)

3-way	354	233	260	186	94	47	26.55%	20.17%
4-way	199	33	186	25	13	8	6.53%	24.24%
5-way	89	19	81	16	8	3	8.99%	15.79%
6-way	40	13	39	12	1	1	2.50%	7.69%
7-way	40	10	40	10	0	0	0.00%	0.00%
8-way	9	1	9	1	0	0	0.00%	0.00%
9-way	4	0	4	0	0	0	0.00%	NA
10-way	4	5	4	5	0	0	0.00%	0.00%
11-way	6	13	6	13	0	0	0.00%	0.00%
12-way	13	13	13	13	0	0	0.00%	0.00%
13-way	12	2	12	2	0	0	0.00%	0.00%
14-way	30	1	30	1	0	0	0.00%	0.00%
15-way	163	0	163	0	0	0	0.00%	NA

*segment level: with segment as the unit. For one collinear segment identified by method A denoted as S_A and another collinear segment identified by method B denoted as S_B, if S_A and S_B overlapped at least 1 base-pair, S_A and S_B could be considered as equivalent segments. M indicated the results using MAAs as markers, and P indicated that of using protein-coding genes as markers. NA indicated that no segment was identified by the method as denominator. 'specific' indicated that the segments were only identified by the method.

3 Supplementary figure legends

Supplementary Figure S1 Length distribution of MAAs family in 7 chromosomes

Left plot indicated the length distribution of all the MAAs, and right plot indicated the MAAs located in individual chromosome.

Supplementary Figure S2 MAAs (left) vs protein-coding genes (right) length distribution in Cucumber

Red curve indicated the normal distribution and green indicated the MAAs (left) and gene (right) density distribution.

Supplementary Figure S3 GC content of MAAs located in different genomic features

Red line as mean GC content of MAAs, blue as mean GC content of Cucumber genome. cds: coding sequences; utr3: 3' untranslated region; utr5: 5' untranslated region; NC: noncoding RNA.

Supplementary Figure S4 Evaluation of MAAs and genes locus uniform distribution using KS.test (Chromosome 3 as case)

Hollow circle indicating the 1000 times of D-statistic values produced by KS.test; full curve indicating the density of MAAs or gene across the chromosome 3. Group1 (red hollow circles): Protein-coding genes vs uniform dataset with the same number of protein-coding genes; Group2 (blue hollow circles): MAAs vs uniform dataset with the same number of MAAs; Group3 (green hollow circles): uniform with genes vs uniform with MAAs; D-statistic values were plotted with 1000 times of random experiment. Uniform datasets were produced by runif function in R.

Supplementary Figure S5 Distribution of n-way (top) and 2way-n (bottom) collinear segments (protein-coding genes-based) among 15 angiosperms plants

The n-way($n \in \{3,4, \cdots, 15\}$) collinear segments indicated the species group that contained cucumber and other related species which were gradually incorporated along the topology of species tree in Figure 1 with cucumber as origin. It could be considered as the multiple species level of collinear segments; 2way-d($d \in \{2,3,\cdots,15\}$, d as the species index) collinear segments indicated the pairwise alignment for each of other 14 species indexed by d, where d was gradually increased with the divergence from the cucumber according to the phylogenetic tree (Figure 1). Thus, different d represents *Cucumis melo, Citrullus lanatus, Malus domestica, Glycine max, Populus trichocarpam, Citrus sinensis, Brassica rapa, Arabidopsis thaliana, Arabidopsis lyrata, Vitis vinifera, Solanum tuberosum, Setaria italic, Brachypodium distachyon and Oryza brachyantha*, respectively.

Supplementary Figure S6 The Jaccard coefficient of 2way-d collinear segments by two methods (MAAs-based and protein-based)

Left: base level, with the base pair as the unit, Right: segments level, with the segment as the unit. For one collinear segments identified by method A denoted as S_A and another collinear segments identified by method B denoted as S_B, if S_A and S_B overlapped at least 1 base-pair, S_A and S_B could be considered as equivalent segments.

Supplementary Figure S7 Annotation results comparison among 8 different pipelines

opp2go (MAAs):our pipeline using MAAs as genomic markers, opp2go(proteins): our pipeline using protein-coding genes as genomic markers, b2go:Blast2GO, ips2go:InterProScan pipeline, orthomcl2go:OrthoMCL pipeline, trib2go:Trinotate-Blast, trip2go:Trinotate-pfam and uniprot:Uniprot resource. Top-left indicated the Jaccard similarity for BP subset (structure-free); Top-right indicated the Jaccard similarity for CC subset (structure-free); Bottom-left indicated the sematic similarity for CC subset (structure-based).

4 Additional files list

Additional file 1: Table S3. All the MAAs information with cucumber as reference

Additional file 2: Table S4. Results by Opp2GO(MAAs-based) pipeline

Additional file 3: Table S5. Results by Opp2GO(protein-based) pipeline

Additional file 4: Table S6. Results by Blast2GO pipeline

Additional file 5: Table S7. Results by InterPro2GO pipeline

Additional file 6: Table S8. Results by OrthoMCL pipeline

Additional file 7: Table S9. Results by Trinotate-Blast pipeline

Additional file 8: Table S10. Results by Trinotate-pfam pipeline

Additional file 9: Table S11. OPPs_inference_results (MAAs-based)

Additional file 10: Table S12. OPPs_inference_results (protein-based)

Additional file 11: Table S13. OPSS_score_results

Additional file 12: Table S14. PO_results

Additional file 13: Table S15. Results by Uniprot pipeline

5 References

Conesa, A., Gotz, S., Garcia-Gomez, J.M., Terol, J., Talon, M., and Robles, M. (2005). Blast2GO: a universal tool for annotation, visualization and analysis in functional genomics research. *Bioinformatics* 21(18)**,** 3674-3676. doi: 10.1093/bioinformatics/bti610.

Finn, R.D., Bateman, A., Clements, J., Coggill, P., Eberhardt, R.Y., Eddy, S.R., et al. (2014). Pfam: the protein families database. *Nucleic Acids Res* 42(Database issue)**,** D222-230. doi: 10.1093/nar/gkt1223.

Finn, R.D., Clements, J., Arndt, W., Miller, B.L., Wheeler, T.J., Schreiber, F., et al. (2015). HMMER web server: 2015 update. *Nucleic Acids Res* 43(W1)**,** W30-38. doi: 10.1093/nar/gkv397.

Grabherr, M.G., Haas, B.J., Yassour, M., Levin, J.Z., Thompson, D.A., Amit, I., et al. (2011). Full-length transcriptome assembly from RNA-Seq data without a reference genome. *Nat Biotechnol* 29(7)**,** 644-652. doi: 10.1038/nbt.1883.

Jones, P., Binns, D., Chang, H.Y., Fraser, M., Li, W., McAnulla, C., et al. (2014). InterProScan 5: genome-scale protein function classification. *Bioinformatics* 30(9)**,** 1236-1240. doi: 10.1093/bioinformatics/btu031.

Li, L., Stoeckert, C.J., Jr., and Roos, D.S. (2003). OrthoMCL: identification of ortholog groups for eukaryotic genomes. *Genome Res* 13(9)**,** 2178-2189. doi: 10.1101/gr.1224503.
